# Decoding the Therapeutic Effects of Acupuncture in Hemorrhagic Stroke Using Single‐Cell RNA Sequencing

**DOI:** 10.1002/cns.70689

**Published:** 2025-12-09

**Authors:** Chen Ruan, Jia Du, Wentao Zhang, Jiacheng Song, Peipei Feng, Jiajun Shi, Kelang Lou, Yuqiang Lu, Xinwei Li, Zhongwei Guo, Hao Liu

**Affiliations:** ^1^ Tongde Hospital of Zhejiang Province Affiliated to Zhejiang Chinese Medical University (College of Integrated Traditional Chinese and Western Medicine Clinical Medicine) Hangzhou China; ^2^ Zhejiang Academy of Traditional Chinese Medicine Hangzhou China

**Keywords:** acupuncture, APOE‐TREM2 signaling, hemorrhagic stroke, microglia, single‐cell RNA sequencing

## Abstract

**Background:**

Acupuncture has been proposed as a therapeutic intervention for stroke recovery, yet the underlying molecular mechanisms remain poorly understood.

**Method:**

In this study, we used a mouse model of hemorrhagic stroke induced by autologous blood injection to investigate the effects of acupuncture on post‐stroke recovery at the cellular and molecular levels, utilizing single‐cell RNA sequencing.

**Results:**

Our findings revealed that acupuncture modulates the gene expression of microglia, astrocytes, and oligodendrocytes, three major glial cell types, which may contribute to the improvement of stroke‐induced phenotypes. Notably, we identified a potential role of the APOE‐TREM2 signaling axis, with ligand‐binding interactions enhancing microglia activation and promoting their neuroprotective functions. These findings also suggested that acupuncture may promote microglia–astrocyte interactions, leading to enhanced neuroinflammation resolution and tissue repair.

**Conclusions:**

Our study provided new insights into the cellular mechanisms underlying acupuncture's therapeutic effects in stroke recovery and highlighted the potential of targeting glial cell‐mediated pathways, including APOE‐TREM2, as a strategy for improving post‐stroke rehabilitation.

## Graphical abstract

The graphical abstract delineates the mechanisms by which acupuncture mitigates the effects of hemorrhagic stroke through the modulation of glial cells. Central to these mechanisms are APOE‐TREM2‐mediated microglial activation, astrocyte‐mediated neuroprotection, and oligodendrocyte‐driven myelination, which collectively enhance functional recovery. Single‐cell RNA sequencing provides insights into the cell‐type‐specific transcriptional alterations that underpin these effects. The design visually underscores the synthesis of cellular, molecular, and behavioral data, thereby highlighting the therapeutic potential of acupuncture in stroke rehabilitation.

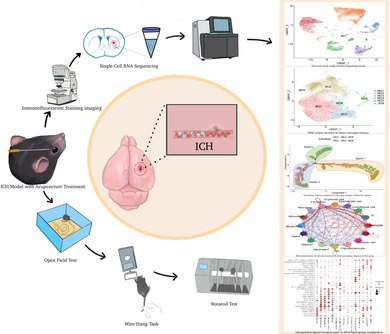

## 1 Introduction

Stroke remains one of the leading causes of death and long‐term disability globally, with hemorrhagic stroke accounting for approximately 15% of cases [1, 2]. Hemorrhagic stroke results from the rupture of cerebral blood vessels, leading to intracerebral hemorrhage, neuronal death, inflammation, and disruption of normal brain function [1, 3]. The pathophysiological processes involved in hemorrhagic stroke include excitotoxicity, oxidative stress, and neuroinflammation, which exacerbate tissue injury and worsen neurological outcomes [3, 4]. Despite the development of therapeutic interventions such as surgical hematoma evacuation and medical management strategies to control intracranial pressure, these treatments are limited in efficacy and do not directly promote long‐term recovery [5, 6]. Furthermore, these interventions do not address the long‐term complications of stroke, such as neuronal dysfunction and cognitive impairment, underscoring the need for novel therapeutic strategies to promote recovery.

Acupuncture, a therapeutic modality rooted in Traditional Chinese Medicine, has been used for centuries to treat various ailments, including neurological disorders such as stroke [7, 8]. Increasing evidence from clinical and experimental studies suggests that acupuncture may have beneficial effects on post‐stroke recovery by improving motor function, reducing neurological deficits, and enhancing overall recovery [9, 10, 11]. The mechanisms by which acupuncture exerts its effects are multifaceted, involving modulation of neural, vascular, and immune systems [12]. Acupuncture has been shown to improve cerebral blood flow, reduce neuroinflammation, promote neurogenesis, and enhance synaptic plasticity, all of which contribute to its potential as a therapeutic intervention for stroke [13, 14]. Despite the growing body of evidence supporting acupuncture's efficacy in stroke rehabilitation, the precise molecular mechanisms underlying its effects remain poorly understood. Understanding these mechanisms at the molecular and cellular levels is critical to developing more targeted and effective therapies for stroke patients.

The advent of single‐cell RNA sequencing (scRNA‐seq) has revolutionized the study of complex biological processes by enabling the analysis of gene expression at the resolution of individual cells. ScRNA‐seq provides a detailed view of cellular heterogeneity and allows researchers to identify specific molecular signatures associated with different cell types in response to disease or therapeutic interventions [15, 16]. In the context of stroke, scRNA‐seq has been applied to investigate the gene expression changes in various cell types, including neurons, glial cells, and endothelial cells, following hemorrhagic injury [17, 18]. This approach has provided valuable insights into the molecular pathways involved in stroke pathophysiology, such as neuroinflammation, cell survival, and repair processes. Furthermore, scRNA‐seq offers a powerful tool to study the cellular and molecular effects of acupuncture on stroke recovery. By analyzing transcriptomic changes in response to acupuncture in a stroke model, it is possible to gain a deeper understanding of how acupuncture modulates gene expression across different cell types in the brain, including neurons, astrocytes, microglia, and endothelial cells. This approach can uncover new molecular targets and biomarkers for acupuncture's therapeutic effects, potentially improving the precision of acupuncture‐based interventions.

To study the effects of acupuncture on stroke recovery, animal models are essential for simulating the pathophysiological processes of hemorrhagic stroke and evaluating the impact of therapeutic treatments. One widely used model for inducing hemorrhagic stroke is the autologous blood injection (ABI) model, where hemorrhage is induced by the intracerebral injection of autologous blood, creating a localized hematoma [17, 19]. This model mimics human hemorrhagic stroke by generating a focal area of brain hemorrhage and is particularly useful for studying the cellular and molecular changes that occur in the brain following stroke [20, 21]. The ABI model has been widely used to investigate the neuroprotective effects of acupuncture, with studies demonstrating that acupuncture can reduce hematoma volume, improve motor function, and promote neuronal survival following stroke [22]. However, the molecular mechanisms by which acupuncture exerts these effects remain unclear, and a more detailed investigation is needed to better understand the cellular and molecular pathways involved.

The primary aim of this study is to investigate the molecular mechanisms underlying acupuncture's effects on stroke recovery using scRNA‐seq. Specifically, we aim to use a mouse model of hemorrhagic stroke induced by autologous blood injection to examine the transcriptomic changes in response to acupuncture treatment. By profiling the gene expression of individual cells in stroke‐affected brain regions, we seek to identify the cellular and molecular pathways that are modulated by acupuncture. We hypothesize that acupuncture may exert its therapeutic effects by modulating neuroinflammation, promoting neurogenesis, enhancing neuronal survival, and improving synaptic plasticity. Furthermore, this study aims to identify potential biomarkers and therapeutic targets for acupuncture in stroke rehabilitation, which could inform the development of more targeted therapies for stroke patients.

Through the application of scRNA‐seq, we aim to provide a comprehensive and cell‐type‐specific view of the molecular changes induced by acupuncture during stroke recovery. This study has the potential to uncover novel insights into the therapeutic mechanisms of acupuncture, which could ultimately lead to more effective strategies for stroke rehabilitation and recovery.

## 2 Methods

### 2.1 Animals

Male C57BL/6J mice, aged 8 weeks (22–25 g; Zhejiang Institute of Traditional Chinese Medicine, Hangzhou, China), were utilized for the study. The animals were housed under a 12‐h light/dark cycle, maintaining a room temperature of 22°C ± 1°C and relative humidity at 50% ± 10%. Prior to surgery, all mice were fasted for 12 h and had water deprivation for 4 h. The study adhered to the Guidelines for the Care and Use of Laboratory Animals as established by the Ministry of Science and Technology of the People's Republic of China in 2006, and all procedures were approved by the Laboratory Animal Welfare and Ethics Committee of Tongde Hospital, Zhejiang Province, China.

### 2.2 ICH Model Establishment

Mice were randomly assigned into three groups: the sham group, the intracerebral hemorrhage (ICH) group, and the ICH with acupuncture treatment group. ICH was induced through the injection of autologous blood, as previously described [23, 24]. Briefly, mice were anesthetized with 3% (vol/vol) isoflurane in a 67%:33% (vol/vol) N_2_O/O_2_ mixture until unresponsive to a tail pinch. They were then placed on a stereotaxic frame and maintained with 1.5% (vol/vol) isoflurane. A heating blanket was used to maintain body temperature between 36°C and 37°C (catalog no. 69000, RWD). A 1‐mm burr hole was drilled on the right cranial bone, 0.8 mm posterior to the bregma and 2.0 mm lateral to the midline. Autologous blood (30 μL) was slowly injected into the right caudate putamen at the coordinates: AP 0.5 mm, L 2.0 mm, and D 3.5 mm. The injection needle was kept in place for 5 min before being removed, and the burr hole was sealed with dental cement and sutures. Sham mice underwent the same surgical procedure, excluding the blood injection. Mice in the acupuncture group received acupuncture treatment following ICH induction.

### 2.3 Acupuncture Treatment

Acupuncture treatment commenced on the first day post‐ICH. Mice underwent two 30‐min sessions of acupuncture daily for seven consecutive days, using 0.25 × 25 mm needles (Huatuo, Suzhou, China). The acupuncture point was GV20, with the needles inserted subcutaneously toward the midpoint between the preauricular point and the lateral canthus of the side affected by the lesion. During each session, the needles were rotated three times for 5 min each at approximately 200 rpm using a custom‐made twirling device.

### 2.4 Magnetic Resonance Imaging (MRI)

MRI imaging was performed on a Bruker 7.0T system (Bruker BioSpin GmbH, Germany) using Turbo RARE T2‐weighted imaging. Imaging parameters were as follows: repetition time (TR) of 2000 ms, echo time (TE) of 36.0 ms, and flip angle of 180°. The field of view (FOV) was set to 1.80 cm, with a matrix size of 256 × 256, resulting in a voxel size of 0.080 × 0.080 mm. A slice thickness of 0.8 mm was used, and a total of 12 slices were acquired. Each scan lasted approximately 4 min and 16 s, with 4 signal averages (NEX) to improve the signal‐to‐noise ratio. The subject was carefully positioned to ensure accurate and stable imaging for detailed anatomical and pathological assessments.

### 2.5 Forelimb Grip Strength Test

The forelimb grip strength test was conducted 7 days post‐ICH using the Grip Strength Meter (XR501, Shanghai Xinruan Information Technology Co. Ltd., Shanghai, China) [25]. Mice were lifted by the tail to grasp a bar mounted on the force gauge. The mice were then slowly pulled away from the meter until the forelimbs released their grip. The maximum forelimb force was recorded by the gauge, and the procedure was repeated three times per mouse, with the average value used for analysis.

### 2.6 Rotarod Test

The rotarod test [26] was performed to assess motor function following ICH. Mice were placed on a rotating rod that gradually accelerated from 0.00027 *g* to 0.027 *g* over 5 min. Each mouse underwent three tests per day with a 5‐min break between tests. The time it took for the mouse to fall off the rod was recorded, and data were expressed as the mean of the three trials.

### 2.7 Open Field Test

Behavioral analysis in an open field was conducted in a square arena (50 × 50 cm) for 10 min [27], with an overhead camera recording the mouse's movements during the day. To monitor nocturnal activity, each mouse was housed individually in a cage with an infrared camera mounted overhead to record its movements for 2 h (11 pm to 1 am). Mouse movements were analyzed using DeepLabCut [28], with 20 frames extracted from each of 8 selected videos. Key body parts (head, anterior thorax, mid‐body, and tail base) were annotated manually, and the neural network was trained for 100,000 iterations. The model's final training and testing errors were 3.37 and 5.75 pixels, respectively, with a detection threshold of 0.6. The trained model was applied to all videos, and Python scripts were used to calculate the total distance traveled and the average speed.

### 2.8 Beam Walking Test

For the beam walking test [29], mice were placed midway on a horizontal rod (70 × 1.5 cm) positioned 30 cm above the ground. The mouse's performance was observed for up to 1 min and scored on a six‐point scale. Each session consisted of three trials.

### 2.9 Wire Hang Task

In the wire hang task [30], mice were placed midway on a wire (50 × 0.1 cm) suspended 50 cm above the ground. Performance was scored on a six‐point scale over a maximum observation time of 1 min, with three trials conducted per session.

### 2.10 Brain Histopathologic Examination

Upon completion of the behavioral tests, mice were euthanized under 0.3% sodium pentobarbital anesthesia. Brain tissue was perfused with 4% paraformaldehyde through the heart. Tissue sections (4 μm) were stained with hematoxylin and eosin for histopathological examination, and cellular structures were observed using a microscope (BX53, Olympus, Tokyo, Japan) at 400× magnification (*n* = 3 animals per condition).

### 2.11 Single Cell RNA Sequencing

Freshly isolated brain tissues from sham, ICH, and acupuncture‐treated mice were stored in ice‐cold Hibernate‐A medium supplemented with 0.5% BSA to maintain cell viability. Tissue dissociation was performed using the Papain Dissociation System (Worthington) at 37°C for 30 min, with gentle pipetting every 5 min. The resulting suspension was filtered through a 40‐μm cell strainer and centrifuged at 300 × *g* for 5 min at 4°C. The pellet was resuspended in PBS with 0.04% BSA, and dead cells were removed using the Dead Cell Removal Kit (Miltenyi Biotec). Viability and concentration were assessed with a hemocytometer and Trypan Blue exclusion, ensuring > 85% viability before proceeding with single‐cell capture.

Single‐cell partitioning and cDNA synthesis were performed using the Chromium Single Cell 3′ Library & Gel Bead Kit v3 (10× Genomics) following the manufacturer's protocol. Single cells were encapsulated into Gel Beads‐in‐Emulsion (GEMs) with reverse transcription reagents using the Chromium Controller. During reverse transcription, mRNA molecules were barcoded with unique UMIs and cell barcodes. The cDNA was then amplified via PCR, purified, and sequencing libraries were constructed through enzymatic fragmentation, adapter ligation, and sample indexing. Libraries were quantified using a Qubit fluorometer (Thermo Fisher) and Bioanalyzer (Agilent) to ensure appropriate size distribution and concentration.

Sequencing was performed on an Illumina platform, with paired‐end reads and a recommended sequencing depth of at least 50,000 reads per cell for sufficient transcriptome coverage. The raw data were processed using the Cell Ranger pipeline (10× Genomics) for demultiplexing, read alignment to the mouse reference genome (10 mm), UMI counting, and cell barcode filtering, resulting in a gene expression matrix for subsequent analysis.

### 2.12 Cellranger Pipeline

The Cell Ranger software was obtained from the 10× Genomics website https://support.10xgenomics.com/single‐cell‐geneexpression/software/downloads/latest. Alignment, filtering, barcode counting, and UMI counting were performed with the cellranger count module to generate a feature‐barcode matrix and determine clusters.

### 2.13 Seurat Pipeline

Cells whose gene number is between 200 and 9000, and mitochondrial gene percentage < 25% were regarded as normal and kept in Seurat v.5 (R package) for following analysis. The filtered gene‐barcode matrix was first normalized using ‘LogNormalize’ methods in Seurat v.5 with default parameters. The top 2000 variable genes were then identified using the ‘vst’ method in Seurat FindVariableFeatures function. Variables ‘nCount_RNA’ and ‘percent.mito’ were regressed out in the scaling step and PCA was performed using the top 2000 variable genes. In parameter settings, the first 50 dimensions of canonical correlation analysis (CCA) and principal‐component analysis (PCA) were used. The “Harmony” method was then used to remove batch effects between samples. Uniform Manifold Approximation and Projection (UMAP) was utilized for dimensionality reduction. Cell clustering was determined using the “FindClusters” function with a resolution of 0.6. We identified conventionally known markers for each cluster. Cell types were then manually assigned and annotated according to conventional cell markers. The FindMarkers function in Seurat v.5 was used to perform differential gene expression analysis. For each cluster, DEGs were generated relative to all of the other cells. A gene was considered significant with avg_log2FC > 0.25 and *p*_val_adj < 0.05.

### 2.14 Functional Enrichment Analysis

Gene ontology (GO) and The Kyoto Encyclopedia of Genes and Genomes (KEGG) enrichment of cluster DEGs was performed using the R package ClusterProfiler (v4.1.0) with Benjamini–Hochberg multiple testing adjustment [31]. GSVA (Gene Set Variation Analysis) was performed by using the R package GSVA, which uses predefined gene sets from the Molecular Signatures Database (MSigDB). The background gene set for GO annotation was the entire genome annotated in the database of org.Hs.eg.db.

### 2.15 Monocle2

The R package Monocle2 (version 2.26.0) was used to calculate the pseudotime trajectories to capture the potential trace of differentiation among different celltypes [32]. With the default settings, the “reduceDimension” and “orderCells” functions were used to infer the cell trajectory.

### 2.16 SCENIC Analysis and Gene Regulatory Network Construction

pySCENIC (version 0.12.1) [33], a computational technique for identifying cell states and generating gene regulatory networks from scRNA‐seq data, was used to infer transcription factors (TFs) in scRNA‐seq data. In brief, a gene co‐expression network was initially built utilizing data from a gene expression matrix. Following that, the “pyscenic ctx” function in the pySCENIC python package was used to find TF‐mRNA regulatory networks with direct regulatory links, and these TFs were classed as regulons. Finally, for each cell corresponding to each regulon, a regulon activity score was calculated, which represents the regulatory activity of the associated regulon in each cell.

### 2.17 Cell–Cell Communication Analysis

The CellChat R package (v1.6.1), a computational framework [34], was used to compare cell–cell communication patterns between Acupuncture and ICH, ICH, and Sham, which contains a curated repository of ligand‐receptor interactions and a statistical framework for inferring lineage‐specific interactions. Hierarchy plot and circle plot embedded were used for visualization of interaction.

### 2.18 Immunofluorescent Staining

Deparaffinize tissue sections by sequential incubation in xylene, ethanol gradients, and finally rinsing with water. Perform antigen retrieval using heat‐induced epitope retrieval (HIER) with 1× citrate buffer (pH 6.0) or EDTA (pH 9.0) for weak targets. For fragile tissues, use microwave retrieval at < 80°C. Block endogenous peroxidase activity with 3% H_2_O_2_, then block nonspecific binding with 10% goat serum. Incubate with primary antibody overnight at 4°C or for 2 h at 37°C, followed by HRP‐conjugated secondary antibody for 1 h at 37°C. Label with TSA reagents (TSA‐570, TSA‐670, TSA‐480, TSA‐520) and wash with PBS between steps. Perform additional antigen retrieval and antibody incubations if required for multiplexing. For nuclear staining, apply DAPI, incubate for 5 min, wash with PBS, and mount with antifade reagent. Store slides at 4°C in the dark. The signal was recorded by using PANNORAMIC MIDI II (3DHISTECH Ltd). The primary or secondary antibodies utilized for staining included Rabbit‐anti‐CD14 (HUABIO, #ET1610‐85), Rabbit‐anti‐TREM2 (Abcam, #ab305103), Rabbit‐anti‐APOE (Proteintech, #18254‐1‐AP), Rabbit‐anti‐IBA1 (Abcam, #ab178847), HRP Goat‐anti‐rabbit IgG (KPL, 074‐1506), and HRP Goat‐anti‐mouse IgG (KPL, 074‐1806).

## 3 Results

### 3.1 Acupuncture as an Effective Therapeutic Approach for Hemorrhagic Stroke Recovery

**FIGURE 1:**
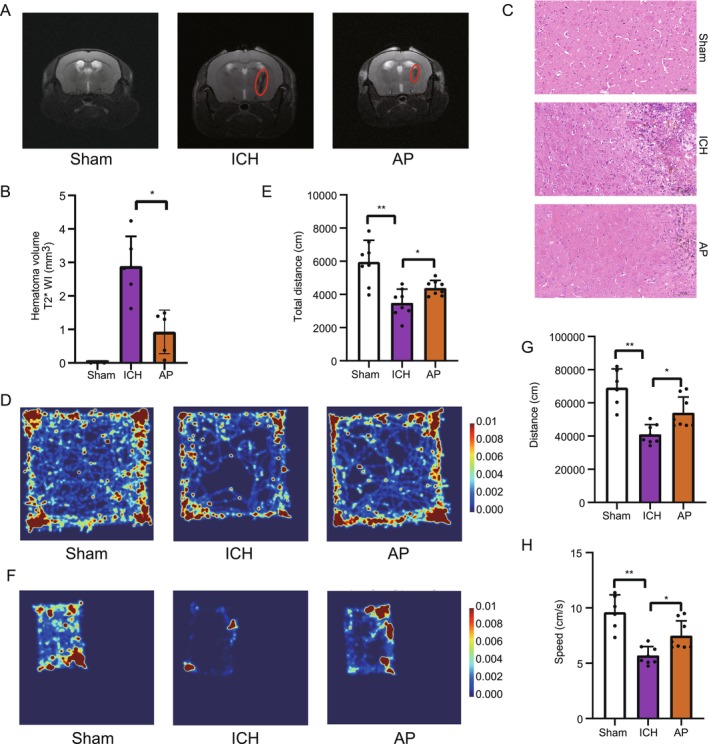
Effects of acupuncture on stroke phenotype in mice. (A) Magnetic resonance imaging (MRI) showing significant reduction in hematoma size in acupuncture‐treated mice compared to stroke model mice. (B) Statistics of hematoma size, related to panel A. *N*: Sham = 3, ICH = 6, AP = 5. (C) Hematoxylin and eosin (HE) staining of brain tissue demonstrating reduced inflammatory responses and tissue damage in the acupuncture group compared to the model group. (D) Daytime open field test for 10 min showing no significant change in the time spent in the center zone but a significant increase in the movement area of the acupuncture group compared to the model group, suggesting improved mobility. (E) Statistics of total movement distance, related to panel D. *N* = 8. (F) Open field test for 2 h at night showing a significant increase in the movement area and speed of the acupuncture group compared to the model group, suggesting improved mobility. (G) and (H) Statistics of movement area and speed, related to panel D. *N*: Sham = 6, ICH = 8, AP = 8.

This study investigates the effects of acupuncture on improving the phenotype of mice with hemorrhagic stroke and explores the underlying biological mechanisms. Various methods were used to characterize and verify the therapeutic effects of acupuncture in alleviating hemorrhagic stroke symptoms. Magnetic resonance imaging (MRI) results demonstrated a significant reduction in hematoma size in mice subjected to acupuncture treatment (Figure 1A,B), while hematoxylin and eosin (HE) staining further confirmed that acupuncture improved the inflammatory microenvironment within the hematoma (Figure 1C). In the open field test, acupuncture did not significantly alter the residence time in the central region, suggesting that these interventions may not strongly influence anxiety‐like behavior in this model. Rather, their primary effects appear to be directed toward enhancing motor recovery and functional repair. This finding underscores the possibility that acupuncture exerts selective rather than broad behavioral benefits, and future studies incorporating additional paradigms of emotional behavior will be important to clarify their impact on affective regulation (Figure 1D,E and Figure S1A). The 2‐h nocturnal open field test further showed that acupuncture‐treated mice exhibited increased movement distance and velocity compared to the model group (Figure 1F–H). Additional behavioral assessments, including the hanging wire test, balance beam test, grip strength test, and rotarod test, consistently demonstrated functional improvements in acupuncture‐treated mice (Figure S1B–E). Furthermore, composite neurological function scores provided additional evidence that acupuncture contributed to the recovery of brain function in hemorrhagic stroke‐afflicted mice (Figure S1F). Collectively, these findings provide strong evidence that acupuncture significantly ameliorates the symptoms of hemorrhagic stroke in mice, offering experimental support for its potential application in hemorrhagic stroke rehabilitation.

### 3.2 Acupuncture Modulates Glial Cell Activity

**FIGURE 2:**
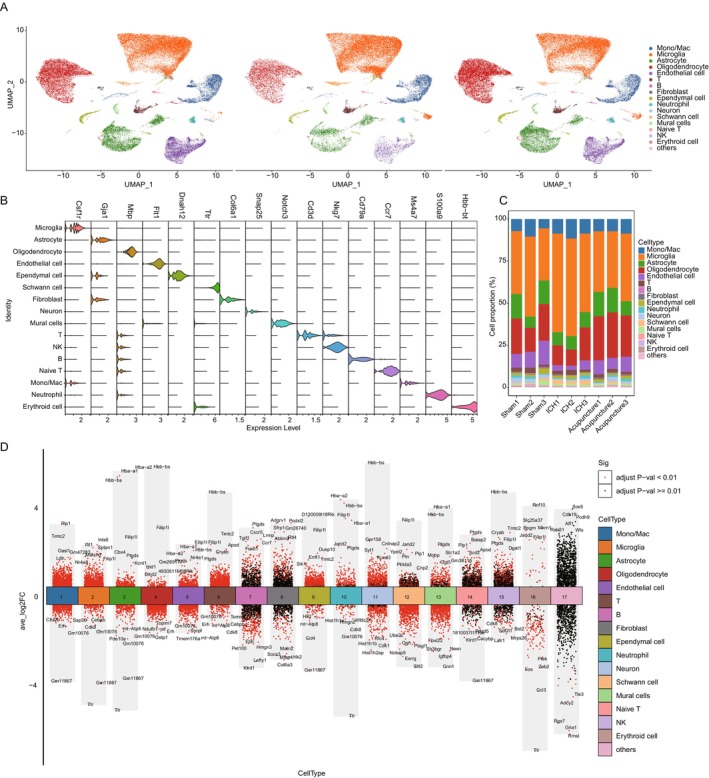
Single‐cell transcriptomic profiling of stroke and acupuncture‐treated mice. (A) Overview of the single‐cell RNA sequencing results, illustrating the identification of 16 distinct cell types across all groups, with a higher proportion of glial cells (microglia, astrocytes, and oligodendrocytes) in the samples from the striatum area around the hematoma. (B) Violin plot of distinct cell markers in different cell types. (C) Cell proportion statistics in sham, ICH and AP‐treated mice. Microglia, astrocyte, and oligodendrocyte were dramatically changed during stroke‐recovery. (D) Volcano plot of differentiated genes in all 16 major cell types of AP‐treated group compared to ICH group.

To further explore the underlying mechanisms, scRNA‐seq was employed to characterize brain tissues from the sham surgery group, model group, and acupuncture‐treated group. Samples were collected from the periphery of the hematoma, anatomically corresponding to the striatum. The sequencing results identified 16 distinct cell types across all three groups, with glial cells—including microglia, astrocytes, and oligodendrocytes—constituting the predominant cell populations, which is consistent with the sampled brain region (Figure 2A,B). While the overall cell type composition remained unchanged among the three groups, the proportions of these key glial cell populations differed significantly (Figure 2C and Table S1). Therefore, the study focused on the roles of microglia, astrocytes, and oligodendrocytes in the acupuncture‐mediated recovery process. Gene expression analysis revealed significant transcriptional changes between the sham and model groups. For instance, genes such as Gm11867, Gm10076, and Tomm7 were upregulated across multiple cell types in the model group, indicating an altered cellular response likely associated with post‐hemorrhagic stroke inflammation and neural damage (Figure S2). Interestingly, acupuncture treatment resulted in a marked reversal of these pathological changes, with notable alterations in glial cell gene expression profiles. Compared to the model group, acupuncture‐treated astrocytes and oligodendrocytes exhibited significant upregulation of Hbb‐bs, Hba‐a1, and Hba‐a2, suggesting a potential role in neuroprotection and repair mechanisms (Figure 2D). The upregulation of these genes may be associated with enhanced oxygen transport, antioxidative responses, and metabolic support in glial cells, which could contribute to the observed functional improvements in hemorrhagic stroke‐affected mice. These findings suggest that acupuncture facilitates post‐hemorrhagic stroke recovery through modulation of glial cell function, thereby promoting neuroprotection, reducing inflammation, and enhancing neural repair.

### 3.3 Microglial Response to Acupuncture and Its Role in Hemorrhagic Stroke Recovery

Microglia, the resident immune cells of the central nervous system (CNS), play a crucial role in neuroinflammation and tissue repair following hemorrhagic stroke [35]. Previous studies have demonstrated that microglia rapidly respond to brain injury, undergoing morphological and transcriptional changes that contribute to both neuroprotection and secondary damage, depending on the context and activation state [36, 37]. In the acute phase of hemorrhagic stroke, activated microglia contribute to the clearance of cellular debris and secrete pro‐inflammatory cytokines such as TNF‐α, IL‐1β, and IL‐6, which can exacerbate neuronal injury if persistently elevated [38, 39]. However, in later phases, microglia transition toward a reparative phenotype, characterized by anti‐inflammatory signaling, neurotrophic factor secretion, and support for remyelination and synaptic remodeling [40]. Given their dual roles, modulating microglial activity has been proposed as a potential therapeutic strategy for hemorrhagic stroke recovery.

**FIGURE 3:**
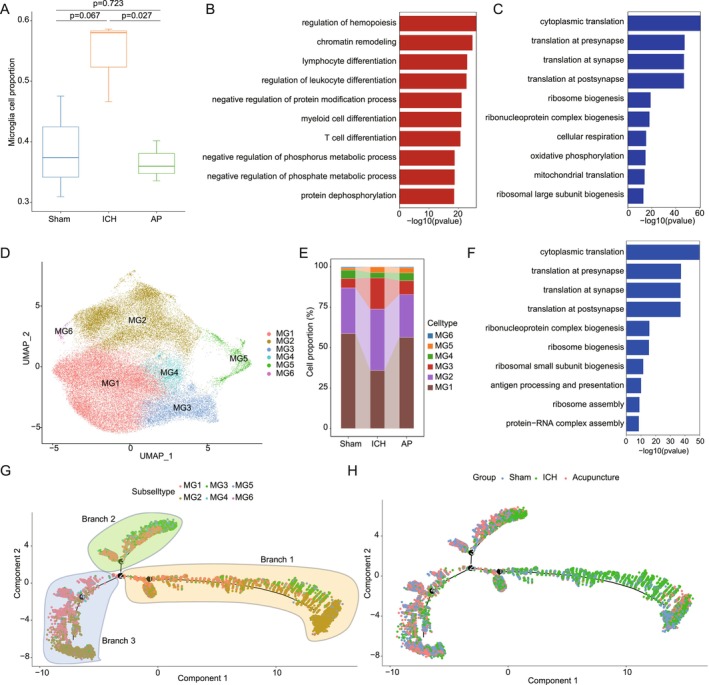
Role of microglia in acupuncture‐mediated stroke recovery. (A) Changes in microglial cell proportion between the sham, ICH, and AP groups. (B) Upregulated Gene Ontology (GO) term at microglia in AP group compared to ICH group. (C) Downregulated GO term at microglia in AP group compared to ICH group. (D) UMAP analysis identifies six distinct microglial subtypes. (E) Significant proportional changes in MG1 and MG3 subtypes between the sham, ICH, and AP groups. (F) Downregulated GO term at microglia subtype MG1 in AP group compared to ICH group. (G) Pseudotime trajectory analysis of 6 microglia subtypes, labeled by subtypes. (H) Pseudotime trajectory analysis of 6 microglia subtypes, labeled by groups, showing MG1 and MG3 diverge along distinct branches (Branches 1 and 3), implicating subtype‐specific roles in recovery.

In this study, we analyzed microglial dynamics in response to acupuncture treatment. Compared to the sham group, the hemorrhagic stroke model group exhibited a significant increase in microglial proportion, aligning with the well‐documented microglial activation following hemorrhagic brain injury (Figure 3A). Notably, acupuncture treatment led to a partial reduction in microglial numbers, suggesting a potential normalization of microglial reactivity (Figure 3A). This decrease in microglial abundance correlates with improved hemorrhagic stroke recovery, indicating that acupuncture may facilitate the resolution of neuroinflammation and promote a shift toward a reparative microglial phenotype. The GO enrichment analysis revealed distinct transcriptional changes in microglia across different experimental groups. In the model group, as shown in Figure S3A, upregulated pathways included cytoplasmic translation, translation at synapse, ribosome assembly, and energy derivation by oxidation of organic compounds, reflecting an overall increase in microglial metabolic activity and protein synthesis, which may be associated with their activated state in response to injury. Conversely, pathways such as response to virus, regulation of hemopoiesis, positive regulation of cellular catabolic process, and myeloid cell differentiation were downregulated, suggesting an impaired immune regulatory function and disrupted hematopoietic signaling (Figure S3B). These findings indicate that microglia in the hemorrhagic stroke model group exhibit a predominantly pro‐inflammatory profile, potentially contributing to sustained neuroinflammation and secondary damage.

Acupuncture treatment significantly altered microglial transcriptional profiles. Compared to the model group, as shown in Figure 3B, the acupuncture‐treated group exhibited upregulation of pathways associated with regulation of hemopoiesis, chromatin remodeling, and lymphocyte differentiation, suggesting a potential enhancement of neuroimmune homeostasis and epigenetic reprogramming of microglial function. Notably, pathways related to cytoplasmic translation, translation at presynapse, and ribonucleoprotein complex biogenesis were downregulated, indicating a shift away from the highly activated microglial state observed in the model group (Figure 3C). These findings suggest that acupuncture may facilitate the transition of microglia toward a neuroprotective phenotype, thereby mitigating excessive neuroinflammation and promoting neural repair. Additionally, when comparing the acupuncture group to the sham group, pathways such as spindle elongation, regulation of mitotic nuclear division, and mitotic nuclear division were upregulated, while immune‐related pathways such as defense response to virus, response to interferon‐beta, and response to type II interferon were downregulated (Figure S3C,D). These results suggest that acupuncture may enhance microglial proliferation and promote CNS homeostasis while suppressing aberrant immune activation, which could contribute to improved hemorrhagic stroke recovery outcomes.

### 3.4 Microglial Subtype‐Specific Responses to Acupuncture

Given the heterogeneity of microglial populations, we further examined transcriptional changes at the microglial subtype level. Unsupervised clustering and UMAP visualization revealed six distinct microglial subpopulations across all three experimental groups (Figure 3D). Notably, MG1 and MG3 subtypes exhibited significant changes following acupuncture treatment, suggesting their potential involvement in hemorrhagic stroke recovery (Figure 3E).

MG3, which demonstrated pronounced alterations post‐acupuncture, exhibited downregulation of pathways related to cytoplasmic translation, translation at presynapse, and antigen processing and presentation (Figure 3F). The suppression of these pathways suggests reduced pro‐inflammatory and antigen‐presenting activity in MG3 following acupuncture, which may contribute to the attenuation of neuroinflammation. This finding aligns with previous studies showing that excessive antigen presentation by microglia can exacerbate neuroinflammation in hemorrhagic stroke, and therapies that limit this process may confer neuroprotection [41].

In contrast, MG1 displayed a distinct transcriptional response to acupuncture. As shown in Figure S3E, pathways such as regulation of developmental growth, regulation of GTPase activity, and response to peptide hormone were upregulated in MG1, suggesting a potential role in neurogenesis, cellular signaling, and neurotrophic support. Meanwhile, translation at presynapse, ribonucleoprotein complex biogenesis, and ribosomal small subunit assembly were downregulated, indicating a shift away from the hyperactive translational state observed in the model group (Figure S3F). These findings suggest that MG1 may contribute to neural repair by supporting synaptic remodeling and metabolic homeostasis.

To further elucidate the functional trajectories of these microglial subtypes, we performed pseudotime trajectory analysis (Figure 3G,H), which revealed that MG3 primarily exhibited significant changes along branch 1, whereas MG1 showed prominent alterations along branch 3. This suggests that MG3 and MG1 may be involved in distinct phases of acupuncture‐mediated hemorrhagic stroke recovery, with MG3 potentially playing a role in the early resolution of neuroinflammation and MG1 contributing to later‐stage repair and regeneration.

Collectively, these findings highlight the complex and dynamic nature of microglial responses to acupuncture. By modulating microglial activation states and subtype‐specific functions, acupuncture appears to promote a more balanced immune response, reduce detrimental inflammation, and support neural repair mechanisms.

### 3.5 The Role of Astrocytes in Acupuncture‐Induced Recovery in Hemorrhagic Stroke Mice

Astrocytes play a critical role in maintaining homeostasis within the CNS by regulating neurotransmitter recycling, blood–brain barrier integrity, synaptic plasticity, and immune responses [42, 43]. They are highly dynamic and respond to neural injury by undergoing reactive astrogliosis, which involves changes in gene expression, proliferation, and morphology [44, 45]. This process can be neuroprotective or neurotoxic, depending on the context and severity of the injury [46]. In hemorrhagic stroke, astrocytes are known to contribute to both neuronal recovery and secondary injury [43]. Initially, they support neuronal survival by releasing neurotrophic factors and scavenging reactive oxygen species [47, 48]. However, prolonged activation can lead to the formation of a glial scar, which can impede axonal regeneration and limit functional recovery. Given these dual roles, understanding astrocyte behavior in response to acupuncture is crucial for elucidating its therapeutic mechanisms.

**FIGURE 4:**
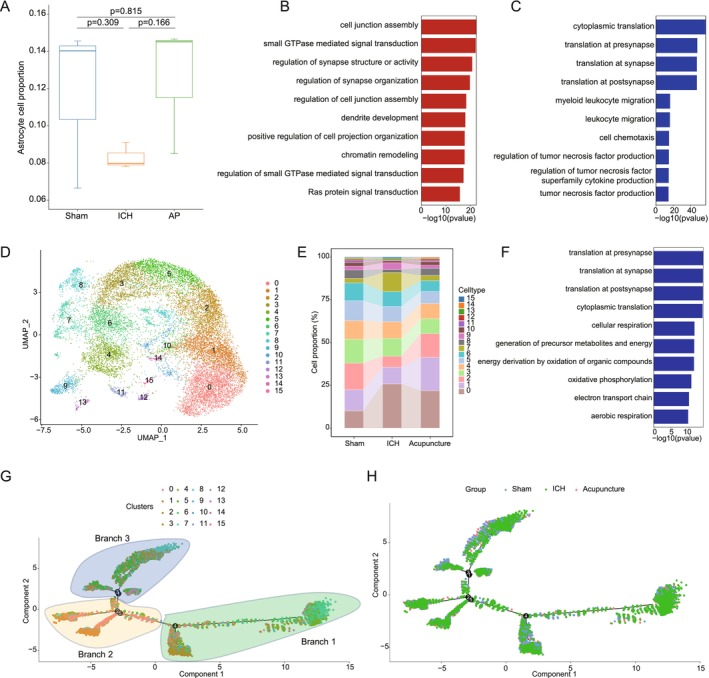
Astrocytic heterogeneity and functional shifts in response to acupuncture. (A) Changes in astrocyte cell proportion between the sham, ICH, and AP groups. (B) Upregulated GO term at astrocyte in AP group compared to ICH group. (C) Downregulated GO term at astrocyte in AP group compared to ICH group. (D) UMAP analysis identifies 16 distinct astrocytes' subtypes. (E) Significant proportional changes in Subclass 0 and Subclass 7 astrocyte subtypes between the sham, ICH, and AP groups. (F) Downregulated GO term at astrocyte subtype Subclass 0 in AP group compared to ICH group. (G) Pseudotime trajectory analysis of 16 microglia subtypes, labeled by subtypes. (H) Pseudotime trajectory analysis of 16 microglia subtypes, labeled by groups, showing Subclass 0 and Subclass 7 diverge along distinct branches, implicating subtype‐specific roles in recovery.

To investigate astrocytic changes following acupuncture treatment in hemorrhagic stroke mice, we analyzed astrocyte populations before and after acupuncture. As shown in Figure 4A, compared to the sham group, the proportion of astrocytes was significantly reduced in the stroke model group, whereas acupuncture treatment partially restored astrocyte numbers, indicating a potential role in phenotype recovery. The GO enrichment analysis revealed that astrocytes in the model group exhibited upregulation of pathways associated with the regulation of inflammatory responses, myeloid leukocyte migration, and leukocyte migration, while neuron migration, forebrain development, and pallium development pathways were downregulated (Figure S4A,B). These findings suggest that hemorrhagic stroke induces a neuroinflammatory environment that disrupts neurodevelopmental processes. In contrast, acupuncture upregulated pathways related to cell junction assembly, small GTPase‐mediated signal transduction, and the regulation of synapse structure or activity while downregulating cytoplasmic translation, translation at presynapse, and myeloid leukocyte migration (Figure 4B,C). This suggests that acupuncture may promote synaptic connectivity and structural reorganization while mitigating excessive neuroinflammation. Additionally, a comparison between the acupuncture and sham groups revealed that acupuncture increased the expression of pathways involved in cell junction assembly, synapse assembly, and positive regulation of cell junction assembly while downregulating cytoplasmic translation, oxidative phosphorylation, and cellular respiration (Figure S4C,D). These results indicate that acupuncture may enhance synaptic stabilization and cell–cell communication while reducing metabolic stress in astrocytes.

### 3.6 Astrocyte's Subtype‐Specific Responses to Acupuncture

To further explore astrocyte heterogeneity in response to acupuncture, we performed single‐cell clustering and identified 16 astrocyte subtypes, a higher degree of classification than observed in microglial populations (Figure 4D). This suggests greater functional diversity among astrocytes, which may be critical for stroke recovery. Notably, subclass 0 and subclass 7 exhibited the most significant changes following acupuncture (Figure 4E). In subclass 0, acupuncture resulted in a significant downregulation of pathways related to cytoplasmic translation, cellular respiration, and the generation of precursor metabolites and energy, indicating a shift away from a highly metabolically active state, possibly reducing cellular stress and promoting repair (Figure 4F). In contrast, subclass 7 exhibited upregulation of pathways associated with cell junction assembly, regulation of developmental growth, and the Wnt signaling pathway, suggesting enhanced structural remodeling and neurodevelopmental support (Figure S4E). Conversely, pathways related to translation at the presynapse, protein‐RNA complex assembly, and non‐membrane‐bounded organelle assembly were downregulated, which may indicate a shift in astrocyte function from active translation toward structural stabilization (Figure S4F). Furthermore, as shown in Figure 4G,H, pseudotime analysis revealed that subclass 0 primarily exhibited significant changes in branch 3, while subclass 7 showed changes in both branch 1 and branch 3, suggesting that these astrocyte subtypes play distinct but complementary roles in acupuncture‐induced recovery in hemorrhagic stroke mice.

### 3.7 Oligodendrocytes and Their Role in Stroke Recovery Under Acupuncture

Oligodendrocytes are the myelinating cells of the CNS, playing a crucial role in maintaining axonal integrity, promoting neuronal function, and regulating synaptic plasticity [49]. Beyond myelination, oligodendrocytes provide metabolic support to neurons, modulate inflammatory responses, and contribute to CNS repair after injury [4]. Previous studies have demonstrated that oligodendrocytes are highly sensitive to brain injury, undergoing apoptosis due to excitotoxicity, oxidative stress, and inflammatory cytokines [50]. In animal models of hemorrhagic stroke, oligodendrocyte loss is accompanied by myelin degradation, leading to impaired neuronal communication and functional deficits [4, 18]. However, oligodendrocyte precursor cells (OPCs) exhibit a degree of plasticity, proliferating and differentiating into mature oligodendrocytes to restore myelination during recovery [51]. Given these findings, we investigated the dynamic changes in oligodendrocytes before and after acupuncture treatment in a mouse model of hemorrhagic stroke.

**FIGURE 5:**
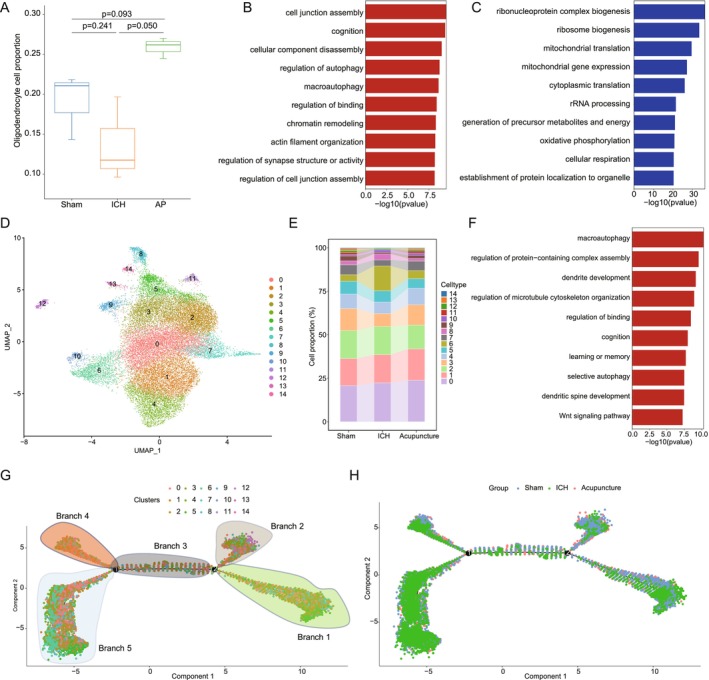
Oligodendrocyte remodeling and myelin repair in acupuncture‐treated stroke. (A) Changes in oligodendrocyte cell proportion between the sham, ICH, and AP groups. (B) Upregulated GO term at oligodendrocyte in AP group compared to ICH group. (C) Downregulated GO term at oligodendrocyte in AP group compared to ICH group. (D) UMAP analysis identifies 15 distinct oligodendrocytes' subtypes. (E) Significant proportional changes in Subclass 3 and Subclass 6 astrocyte subtypes between the sham, ICH, and AP groups. (F) Downregulated GO term at astrocyte subtype Subclass 0 in AP group compared to ICH group. (G) Pseudotime trajectory analysis of 15 oligodendrocyte subtypes, labeled by subtypes. (H) Pseudotime trajectory analysis of 15 oligodendrocyte subtypes, labeled by groups, showing Subclass 3 and Subclass 6 diverge along distinct branches, implicating subtype‐specific roles in recovery.

Our results showed that the proportion of oligodendrocytes was significantly reduced in the model group compared to the sham‐operated group (Figure 5A), suggesting that ischemic injury disrupts oligodendrocyte homeostasis. Interestingly, following acupuncture treatment, the proportion of oligodendrocytes markedly increased, even surpassing that of the sham‐operated group (Figure 5A). This suggests that acupuncture may either enhance oligodendrocyte survival, promote differentiation from OPCs, or facilitate remyelination, potentially contributing to improved neurological function. To explore the molecular mechanisms underlying these changes, we performed GO enrichment analysis. In the model group, as shown in Figure S5A, upregulated pathways included “establishment of protein localization to organelle,” “cellular respiration,” and “protein folding,” suggesting a stress response aimed at restoring protein homeostasis and mitochondrial function. Conversely, as shown in Figure S5B, downregulated pathways included “cell junction assembly,” “gliogenesis,” and “protein dephosphorylation,” indicating impaired cell–cell communication, reduced oligodendrocyte proliferation, and disrupted signaling networks necessary for repair. These results suggest that hemorrhagic stroke leads to substantial dysfunction in oligodendrocytes, limiting their ability to support neuronal recovery.

### 3.8 Acupuncture Modulates Oligodendrocyte Subtypes and Functional Pathways

To further elucidate the effects of acupuncture, we compared the acupuncture‐treated group with the model group and observed significant pathway alterations. Pathways such as “cell junction assembly,” “cognition,” and “cellular component disassembly” were upregulated, indicating enhanced intercellular connectivity, neuronal remodeling, and tissue reorganization (Figure 5B). In contrast, pathways related to “ribonucleoprotein complex biogenesis,” “ribosome biogenesis,” and “mitochondrial translation” were downregulated, suggesting a shift from translational activity toward processes involved in cellular recovery (Figure 5C). We also compared the acupuncture group with the sham‐operated group and found that pathways such as “regulation of cartilage development,” “cell migration involved in sprouting angiogenesis,” and “regulation of chondrocyte differentiation” were upregulated, indicating that acupuncture may promote tissue repair and vascular remodeling (Figure S5C). Meanwhile, pathways related to “regulation of tumor necrosis factor (TNF) production,” “regulation of TNF superfamily cytokine production,” and “TNF production” were downregulated, suggesting that acupuncture may exert an anti‐inflammatory effect on oligodendrocytes (Figure S5D).

To investigate the heterogeneity of oligodendrocytes in response to acupuncture, we performed single‐cell clustering analysis and identified 15 distinct oligodendrocyte subtypes across all groups (Figure 5D), which was notably more than the number of microglial subtypes identified in our previous analysis. This suggests that oligodendrocytes may exhibit greater cellular plasticity in response to ischemic injury and acupuncture treatment. Among these subtypes, subclass 3 and subclass 6 exhibited significant changes after acupuncture (Figure 5E). Specifically, in subclass 6, pathways such as “macroautophagy,” “regulation of protein‐containing complex assembly,” and “dendrite development” were upregulated, suggesting that acupuncture may enhance autophagy‐mediated clearance of damaged cellular components, improve protein complex assembly, and support neuronal repair mechanisms (Figure 5F). In subclass 3, as shown in Figure S5E,F, pathways such as “establishment or maintenance of cell polarity,” “cell junction assembly,” and “protein localization to cell periphery” were upregulated, suggesting enhanced structural organization and cell–cell interactions. In contrast, downregulated pathways in subclass 3 included “cytoplasmic translation,” “oxidative phosphorylation,” and “ribonucleoprotein complex biogenesis,” which may indicate a metabolic shift prioritizing cellular repair over energy‐intensive processes.

Pseudotime trajectory analysis further revealed that subclass 0 primarily exhibited significant changes in branch 3, while subclass 3 and subclass 6 showed dynamic alterations in branches 2 and 5 (Figure 5G,H). This suggests that these two oligodendrocyte subtypes may be particularly involved in the beneficial effects of acupuncture on stroke recovery. Collectively, these findings indicate that acupuncture influences oligodendrocyte function through multiple mechanisms, including enhancing intercellular connectivity, promoting structural remodeling, modulating inflammation, and facilitating cellular repair, all of which may contribute to the overall improvement of neurological function in hemorrhagic stroke.

### 3.9 Microglia Interaction With Other Cell Types During Stroke Recovery Under Acupuncture

Given the established role of microglia in hemorrhagic stroke recovery, this study further investigates microglial interactions with other cell types and how these interactions are modulated by acupuncture treatment. Previous research has demonstrated that microglia, as the resident immune cells of the CNS, are crucial for maintaining homeostasis, responding to injury, and facilitating recovery processes following hemorrhagic stroke [52, 53]. Microglial activation is a hallmark of hemorrhagic stroke, where they transition from a resting to an activated state, potentially leading to neuroinflammation and neuronal damage [54, 55]. However, appropriate modulation of microglial activation has been shown to enhance recovery through the release of neurotrophic factors, regulation of the blood–brain barrier, and phagocytosis of debris [56, 57]. Therefore, it is important to explore how acupuncture affects microglial interactions with other cell types involved in stroke recovery.

In our study, we first examined the changes in cellular interactions in the model group compared to the sham‐operated group. The results showed that both the frequency and intensity of intercellular communication were altered in the model group, with upregulated interactions primarily concentrated between erythroid cells and neutrophils, as well as ependymal cells and neutrophils (Figure S6A–D). This suggests that stroke‐induced inflammation and hemorrhagic injury lead to an increased recruitment of neutrophils, which is consistent with previous findings indicating that neutrophils are one of the first responders to brain injury and contribute to secondary tissue damage through the release of proteases and inflammatory cytokines [58, 59]. Additionally, the downregulated interactions between neutrophils and T cells, as well as neutrophils and monocytes/macrophages, indicate a potential disruption of the normal immune response regulation, which might impair the resolution of inflammation and tissue repair processes in the brain after hemorrhagic stroke.

**FIGURE 6:**
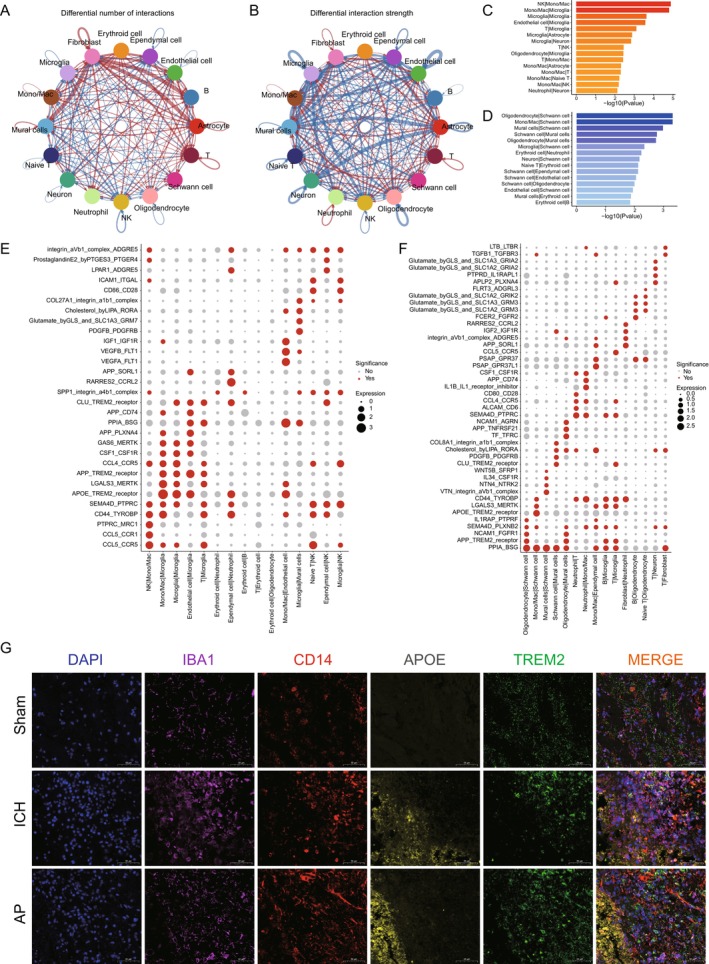
Cell–cell communication networks modulated by acupuncture in stroke recovery. (A) Differential number of cell–cell interaction in AP‐treated group compared to ICH group. (B) Differential cell–cell interaction strength in AP‐treated group compared to ICH group. (C) Upregulated cell–cell interaction pairs in AP‐treated group compared to ICH group. (D) Downregulated cell–cell interaction pairs in AP‐treated group compared to ICH group. (E) Upregulated ligand‐receptor pairs in AP‐treated group compared to ICH group. (F) Downregulated ligand‐receptor pairs in AP‐treated group compared to ICH group. (G) Immunofluorescent staining of IBA1 (microglia marker), CD14 (Mono‐Mac marker), APOE, and TREM2 in sham, ICH, and AP‐treated group.

When comparing the acupuncture group with the model group, as shown in Figure 6A–D, we observed significant changes in cell–cell communication, particularly in the upregulation of interactions between NK cells and monocytes/macrophages (Mono/Mac), as well as between monocytes/macrophages and microglia. These interactions may indicate that acupuncture promotes a coordinated immune response by enhancing the recruitment and activation of monocytes/macrophages and NK cells, which play essential roles in tissue repair, phagocytosis, and neuroprotection. Conversely, the interactions between oligodendrocytes and Schwann cells, as well as between monocytes/macrophages and Schwann cells, were downregulated in the acupuncture group. This could suggest that acupuncture might modulate the differentiation and function of oligodendrocytes and Schwann cells to limit excessive repair processes or to prioritize neuronal regeneration. Overall, these findings highlight the importance of microglia and their interactions with immune and neuronal cells in the recovery process and suggest that acupuncture may help restore or enhance these cell–cell communications for improved brain recovery after hemorrhagic stroke.

### 3.10 Ligand‐Receptor Interactions and Their Modulation by Acupuncture

Next, we focused on the analysis of ligand‐receptor interactions between different cell types in response to acupuncture treatment. Ligand‐receptor interactions play an essential role in cell signaling, regulating numerous cellular processes including inflammation, repair, and neurogenesis. As shown in Figure 6E, in the acupuncture‐treated group, we observed that several ligand‐receptor pairs, such as APOE‐TREM2 and RARRES2‐CCRL2, had stronger interactions compared to the model group. These findings suggest that acupuncture may enhance neuroprotective signaling pathways and promote reparative processes. Specifically, the APOE‐TREM2 interaction has been well‐documented in the context of brain injury and hemorrhagic stroke. APOE, a major apolipoprotein, is involved in lipid metabolism and neuronal repair, while TREM2 is a receptor expressed by microglia and involved in phagocytosis and modulation of neuroinflammation [60, 61]. The activation of the APOE‐TREM2 pathway has been shown to facilitate microglial activation and tissue repair after hemorrhagic stroke [62, 63]. Thus, the enhanced APOE‐TREM2 signaling in the acupuncture group could promote microglial‐mediated repair processes, enhance clearance of cellular debris, and reduce neuroinflammation, ultimately contributing to improved stroke recovery.

In contrast, we also observed a reduction in the interactions of certain ligand‐receptor pairs in the acupuncture group, including PPIA‐BSG and CD44‐TYROBP (Figure 6F). These reductions may indicate a shift in cellular signaling pathways toward those that promote repair and regeneration, rather than pro‐inflammatory pathways. PPIA (peptidylprolyl isomerase A) and BSG (basigin) are involved in cellular adhesion and inflammation, while CD44 is a cell surface glycoprotein involved in cell migration and tissue remodeling [64]. TYROBP (DAP12) is an adapter protein that participates in immune cell signaling, particularly in monocytes/macrophages and microglia [65]. The downregulation of these interactions in the acupuncture group suggests that acupuncture may reduce excessive inflammatory responses, promoting a more balanced immune environment conducive to tissue healing.

Given the well‐established role of the APOE‐TREM2 axis in brain injury and hemorrhagic stroke recovery, we specifically examined the expression of APOE and TREM2 in microglia. As shown in Figure 6G, in the sham‐operated group, the percentage of APOE^+^ cells was low whereas that of TREM2^+^ cells was high. In contrast, the model group exhibited a significantly increased percentage of APOE^+^ cells and a decreased percentage of TREM2^+^ cells (Figure S6E), likely as a result of microglial activation and the attempt to resolve the injury. Interestingly, in the acupuncture group, the percentage of APOE^+^ cells was notably elevated compared to the model group, whereas that of TREM2^+^ cells did not show significant changes (Figure S6E). This suggests that acupuncture may specifically promote APOE‐mediated microglial activation and neuroprotection, which could indicate a selective modulation of the microglial response. The enhanced percentage of APOE4^+^ cells in the acupuncture group aligns with the observed improvements in tissue repair, neurogenesis, and functional recovery, supporting the hypothesis that acupuncture may facilitate a more efficient and controlled microglial response to ischemic injury. Besides, compared with the sham group, the proportion of microglia (IBA1^+^) was significantly increased in the model group, and the proportion of mono_macrophages (CD14^+^) was also markedly elevated (Figure S6E). Both populations showed a significant reduction in the acupuncture group compared with the model group, suggesting that the therapeutic intervention effectively attenuates the pathological activation and expansion of innate immune cell subsets associated with neuroinflammation. In addition, we observed that the proportion of APOE^+^/TREM2^+^ double‐positive cells co‐localized with microglia, as well as those co‐localized with mono_macrophages, was significantly increased from the sham group to the model group, but markedly decreased from the model group to the treatment group. These findings further indicate that the treatment mitigates disease‐associated microglial and macrophage responses, thereby potentially restoring immune homeostasis and limiting neurodegenerative processes.

In summary, the modulation of microglial interactions with other immune and neuronal cells through acupuncture treatment highlights the complexity of cellular communication in hemorrhagic stroke recovery. By enhancing beneficial immune responses and reducing excessive inflammation, acupuncture appears to facilitate an environment that supports tissue repair and regeneration. Furthermore, the selective modulation of ligand‐receptor interactions, such as APOE‐TREM2 signaling, may be a key mechanism by which acupuncture promotes recovery after hemorrhagic stroke. These findings provide valuable insights into the potential therapeutic benefits of acupuncture for stroke patients and underscore the importance of understanding cellular interactions in the context of neuroinflammation and repair.

## 4 Discussion

The present study provides new insights into the molecular mechanisms by which acupuncture may facilitate post‐stroke recovery, with a particular focus on the modulation of glial cells, specifically microglia, astrocytes, and oligodendrocytes. Using a scRNA‐seq approach in a mouse model of hemorrhagic stroke induced by autologous blood injection, we identified significant transcriptional changes in these key glial cell populations following acupuncture treatment. Our findings suggest that acupuncture promotes hemorrhagic stroke recovery at least in part through modulation of glial cell function, although indirect effects mediated by neuron–glia or other intercellular interactions cannot be excluded.

Microglia, the resident immune cells of the CNS, play a critical role in both the acute response to hemorrhagic injury and the resolution of neuroinflammation [36]. In our study, acupuncture treatment was associated with altered gene expression in microglia, with an upregulation of genes involved in neuroprotection, phagocytosis, and cytokine regulation. These changes suggest that acupuncture may enhance microglial activation and promote the clearance of cellular debris, a key step in tissue repair following stroke. While transcriptional profiling and GO analysis suggested that MG1 and MG3 may contribute to distinct phases of recovery, we acknowledge that functional validation (e.g., conditional depletion) was not performed, and thus their causal role in acupuncture efficacy remains to be established. Besides, our findings indicate that the APOE–TREM2 signaling axis plays a critical role in regulating microglial interactions and neuroinflammatory responses. The binding of APOE to the TREM2 receptor is known to enhance microglial activity, particularly in the context of neurodegeneration, by promoting phagocytosis and modulating the inflammatory response [66, 67]. In our study, acupuncture treatment appeared to enhance the APOE–TREM2 pathway, which may contribute to the resolution of neuroinflammation and support tissue regeneration after hemorrhagic stroke. These results are consistent with previous studies showing that the APOE–TREM2 axis is critical for microglial activation in response to injury and disease [68, 69]. Although APOE–TREM2 signaling is well known to modulate microglial function in neurodegenerative diseases such as Alzheimer's disease, its involvement in hemorrhagic stroke may reflect a distinct context. In Alzheimer's disease, this pathway has been primarily implicated in microglial‐mediated amyloid clearance and the regulation of chronic neuroinflammation. By contrast, in hemorrhagic stroke the APOE–TREM2 axis may be engaged in acute immune activation and tissue repair processes triggered by vascular injury and blood extravasation. These differences suggest that while APOE–TREM2 represents a common signaling hub for microglial regulation, the downstream functional outcomes may be disease‐specific, shaped by the temporal dynamics and microenvironmental cues of each pathological condition. Our findings therefore extend the relevance of APOE–TREM2 beyond neurodegenerative settings and highlight its potential as a target for modulating microglial responses in hemorrhagic stroke.

While our transcriptomic analyses suggest that APOE–TREM2 signaling may enhance microglial function during recovery, we did not assess the activation status of downstream pathways such as TYROBP and SYK, nor did we perform functional validation using TREM2 knockout models. Therefore, the causal role of this signaling axis in mediating the efficacy of acupuncture remains to be established. These findings should be interpreted as hypothesis‐generating, providing a foundation for future studies aimed at mechanistically dissecting the APOE–TREM2 pathway and its downstream effectors in the context of hemorrhagic stroke recovery.

Astrocytes, another key glial cell type, have long been known for their role in maintaining the blood–brain barrier and supporting neuronal function under normal and pathological conditions [42]. In hemorrhagic stroke, astrocytes undergo reactive gliosis, which can contribute to both beneficial and detrimental outcomes depending on the context and stage of injury [70]. In this study, we observed significant changes in astrocyte gene expression following acupuncture treatment, with a shift toward a more neuroprotective phenotype. Specifically, acupuncture appeared to modulate the expression of genes involved in astrocytic support of neuronal survival, synaptic plasticity, and regulation of the extracellular matrix. This finding is consistent with previous studies suggesting that astrocytes can promote tissue repair by secreting neurotrophic factors and providing metabolic support to neurons [71]. The modulation of astrocyte function by acupuncture may, therefore, contribute to the overall improvement of neurological function observed in the treated animals.

Oligodendrocytes, the myelinating cells of the CNS, also play a crucial role in recovery following hemorrhagic injury. Myelin damage following stroke can impair neuronal communication and contribute to long‐term disability [18]. In our study, acupuncture treatment was associated with changes in oligodendrocyte gene expression, particularly in pathways related to myelination and oligodendrocyte precursor cell differentiation. These findings suggest that acupuncture may promote remyelination and improve neuronal signaling, which could contribute to functional recovery after hemorrhagic stroke. Previous studies have shown that oligodendrocyte survival and myelination are key factors in the restoration of neural circuits following stroke [72]. Our results, therefore, imply that acupuncture may have a role in supporting oligodendrocyte function and facilitating the repair of damaged neural networks.

Our study also highlights the importance of using scRNA‐seq to investigate the cellular and molecular mechanisms underlying acupuncture's effects in hemorrhagic stroke recovery. Traditional bulk RNA sequencing methods often mask the complexity of cellular responses, whereas scRNA‐seq allows for the identification of specific gene expression changes within individual cell types. By profiling the transcriptomes of microglia, astrocytes, and oligodendrocytes at single‐cell resolution, we were able to uncover previously unrecognized changes in these cell populations following acupuncture treatment. This approach not only enhances our understanding of how acupuncture modulates glial cell function but also provides a powerful tool for identifying potential biomarkers and therapeutic targets for stroke rehabilitation.

There are several key limitations to our study that warrant further exploration. First, while our results suggest that acupuncture modulates glial cell function and promotes recovery, the specific signaling pathways involved in this process remain to be fully elucidated. Further studies are needed to explore the role of additional molecular pathways, such as those related to synaptic plasticity, neuronal survival, and angiogenesis, in mediating acupuncture's therapeutic effects. Second, the scope of our study was limited to a single mouse model of hemorrhagic stroke, and further validation in other stroke models is needed to determine the generalizability of our findings. Finally, while scRNA‐seq provided valuable insights into the gene expression changes in glial cells, it does not provide direct evidence of functional outcomes. Future studies should combine transcriptomic analysis with functional assays, such as electrophysiology and behavioral testing, to better understand how acupuncture impacts neuronal function and recovery.

## 5 Conclusion

Our study provides compelling evidence that acupuncture exerts its therapeutic effects in hemorrhagic stroke recovery through the modulation of glial cell function, particularly microglia, astrocytes, and oligodendrocytes. The APOE‐TREM2 signaling pathway, in particular, appears to play a critical role in enhancing microglial activation and promoting neuroinflammation resolution. These findings contribute to our understanding of how acupuncture may improve post‐stroke recovery at the cellular and molecular levels and provide a foundation for the development of acupuncture‐based therapies targeting glial cells and neuroinflammation in stroke rehabilitation. Future studies are needed to further investigate the signaling networks involved and explore the potential of acupuncture as a therapeutic strategy for enhancing hemorrhagic stroke recovery.

## Author Contributions

H.L. conceived and designed the study and interpreted experiments. C.R. and J.D. performed the experiments and wrote the manuscript. W.Z., J.So., J.Sh. and K.L. performed the experiments. J.So., X.L., Z.G. and Y.L. completed the data processing, normalization, and bioinformatics analyses. All authors have read and approved the manuscript.

## Funding

This study was funded by the National Natural Science Foundation of China (82274648, 81804168); Zhejiang Provincial Natural Science Foundation (LY24H270008); National Leading Medical Specialty Development Project‐Department of Geriatrics, Tongde Hospital of Zhejiang Province ([2024]90662); Zhejiang Provincial Alliance of Traditional Chinese Medicine Advantage Specialty for Geriatric Diseases ([2024]10); State Administration of Traditional Chinese Medicine Science and Technology Department‐Zhejiang Provincial Administration of Traditional Chinese Medicine Co‐construction of Key Laboratory of Research on Prevention and Treatment for depression syndrome [GZY‐ZJ‐SY‐2402].

## Ethics Statement

The animal experiments in this study were approved by the Laboratory Animal Welfare and Ethics Committee of Tongde Hospital of Zhejiang Province, China (approval [2024]011).

## Conflicts of Interest

The authors declare no conflicts of interest.

## Supporting information


**Figure S1:** Additional behavioral assessments of stroke phenotype improvement in acupuncture‐treated mice. (A) Statistics of total time in center area, related to main Figure 1 panel F. *N*: Sham = 6, ICH = 8, AP = 8. (B) Statistics of wire hang task score. *N* = 10. (C) Statistics of beam walking score. *N* = 10. (D) Statistics of grip strength test score. *N* = 11. (E) Statistics of rotarod test score. *N* = 11. (F) Statistics of composite neuroscore. *N* = 10.

**Figure S2:** Quality control and differential gene expression analysis of single‐cell RNA sequencing (scRNA‐seq) data. (A) Quality control scRNA‐seq dataset. (B) Volcano plot of differentiated genes in all 16 major cell types of ICH group compared to sham group.

**Figure S3:** Microglial activation and resolution after acupuncture treatment. (A) Upregulated GO term at microglia in ICH group compared to sham group. (B) Downregulated GO term at microglia in ICH group compared to sham group. (C) Upregulated GO term at microglia in AP‐treated group compared to sham group. (D) Downregulated GO term at microglia in AP‐treated group compared to sham group. (E) Upregulated GO term at microglia subclass MG1 in AP‐treated group compared to ICH group. (F) Downregulated GO term at microglia subclass MG1 in AP‐treated group compared to ICH group.

**Figure S4:** Astrocyte functional plasticity and subtype shifts following acupuncture treatment. (A) Upregulated GO term at astrocyte in ICH group compared to sham group. (B) Downregulated GO term at astrocyte in ICH group compared to sham group. (C) Upregulated GO term at astrocyte in AP‐treated group compared to sham group. (D) Downregulated GO term at astrocyte in AP‐treated group compared to sham group. (E) Upregulated GO term at astrocyte subclass 7 in AP‐treated group compared to ICH group. (F) Downregulated GO term at astrocyte subclass 7 in AP‐treated group compared to ICH group.

**Figure S5:** Oligodendrocyte remodeling and myelin regeneration in acupuncture‐treated stroke mice. (A) Upregulated GO term at oligodendrocyte in ICH group compared to sham group. (B) Downregulated GO term at oligodendrocyte in ICH group compared to sham group. (C) Upregulated GO term at oligodendrocyte in AP‐treated group compared to sham group. (D) Downregulated GO term at oligodendrocyte in AP‐treated group compared to sham group. (E) Upregulated GO term at oligodendrocyte subclass 3 in AP‐treated group compared to ICH group. (F) Downregulated GO term at oligodendrocyte subclass 3 in AP‐treated group compared to ICH group.

**Figure S6:** Alterations in cell–cell communication and ligand‐receptor interactions after acupuncture. (A) Differential number of cell–cell interaction in ICH group compared to sham group. (B) Differential cell–cell interaction strength in ICH group compared to sham group. (C) Upregulated cell–cell interaction pairs in ICH group compared to sham group. (D) Downregulated cell–cell interaction pairs in ICH group compared to sham group. (E) Quantification of APOE and TREM2 positive cell in microglia and mono_macrophage.

**Table S1:** Quantification and statistic analysis of cell types among Sham, ICH, and AP group.

## Data Availability

The data that support the findings of this study are openly available in Genome Sequence Archive at https://ngdc.cncb.ac.cn/gsa/s/6Q85wL1Z, reference number CRA025281.
