# Supplementary figures and images for: Decoding the Therapeutic Effects of Acupuncture in Hemorrhagic Stroke Using Single‐Cell RNA Sequencing
Source: CNS Neurosci Ther. 2025 Dec 9;31(12):e70689. doi: 10.1002/cns.70689 (PMC12686964; doi:10.1002/cns.70689)

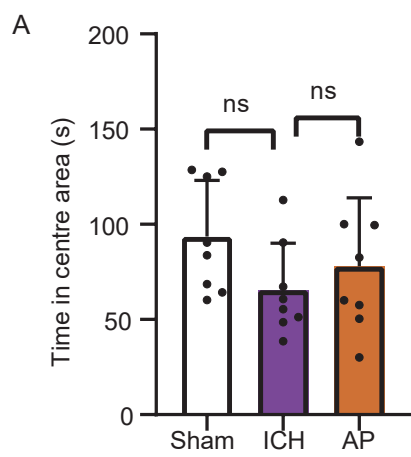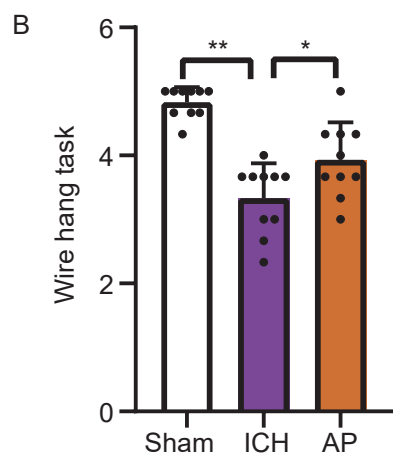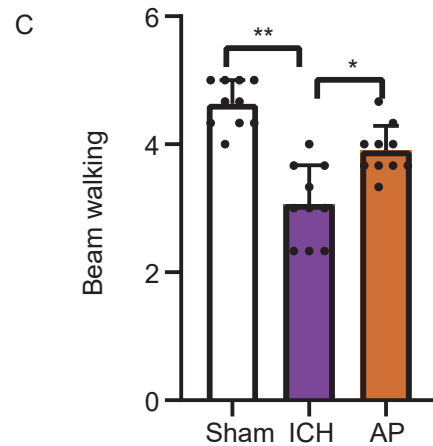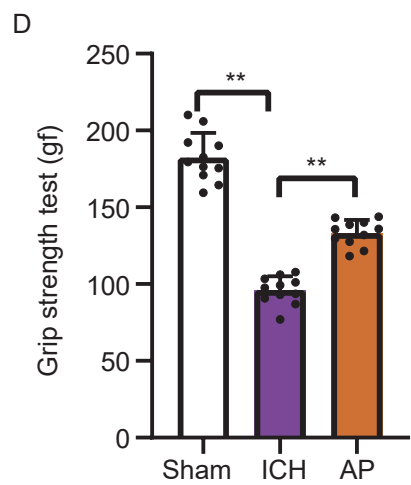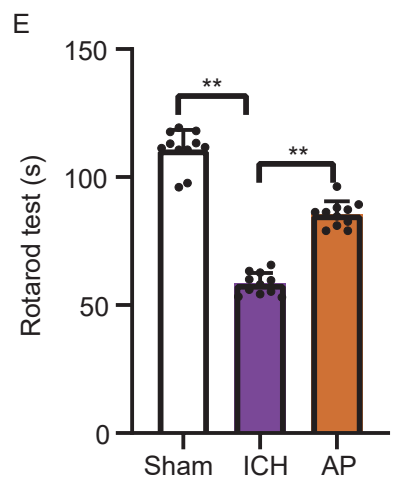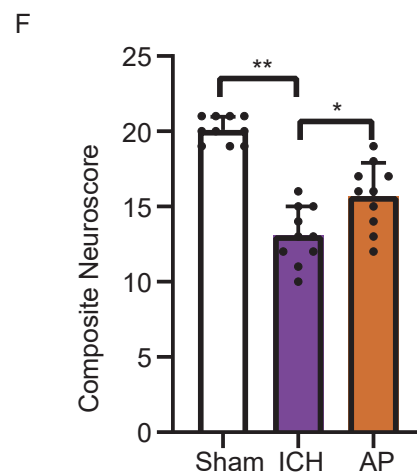

Supplement: Supplementary file 1 — Figure S1: Additional behavioral assessments of stroke phenotype improvement in acupuncture‐treated mice. (A) Statistics of total time in center area, related to main Figure 1 panel F. N: Sham = 6, ICH = 8, AP = 8. (B) Statistics of wire hang task score. N = 10. (C) Statistics of beam walking score. N = 10. (D) Statistics of grip strength test score. N = 11. (E) Statistics of rotarod test score. N = 11. (F) Statistics of composite neuroscore. N = 10. [file CNS-31-e70689-s002.pdf]

A

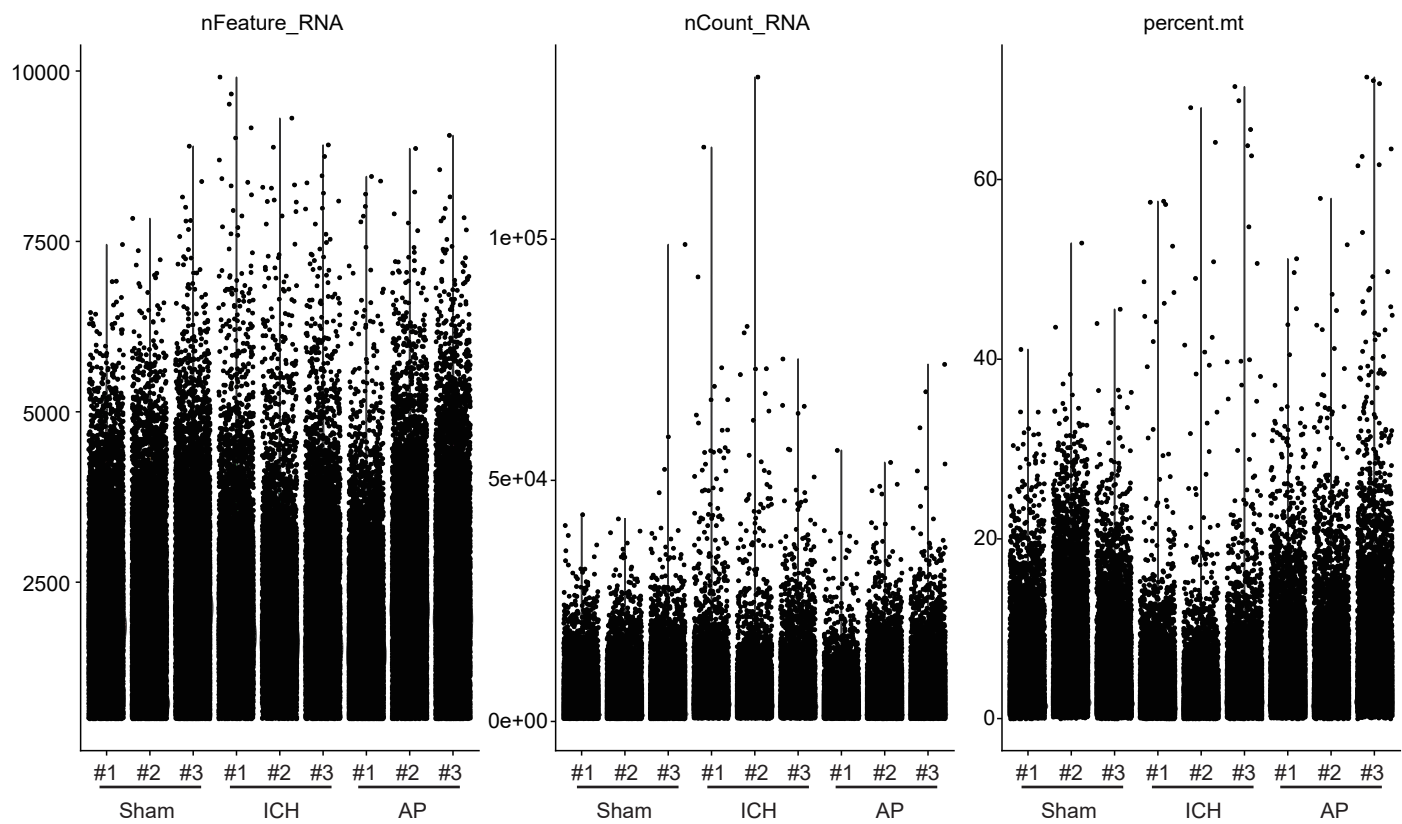

B

ICH vs Sham

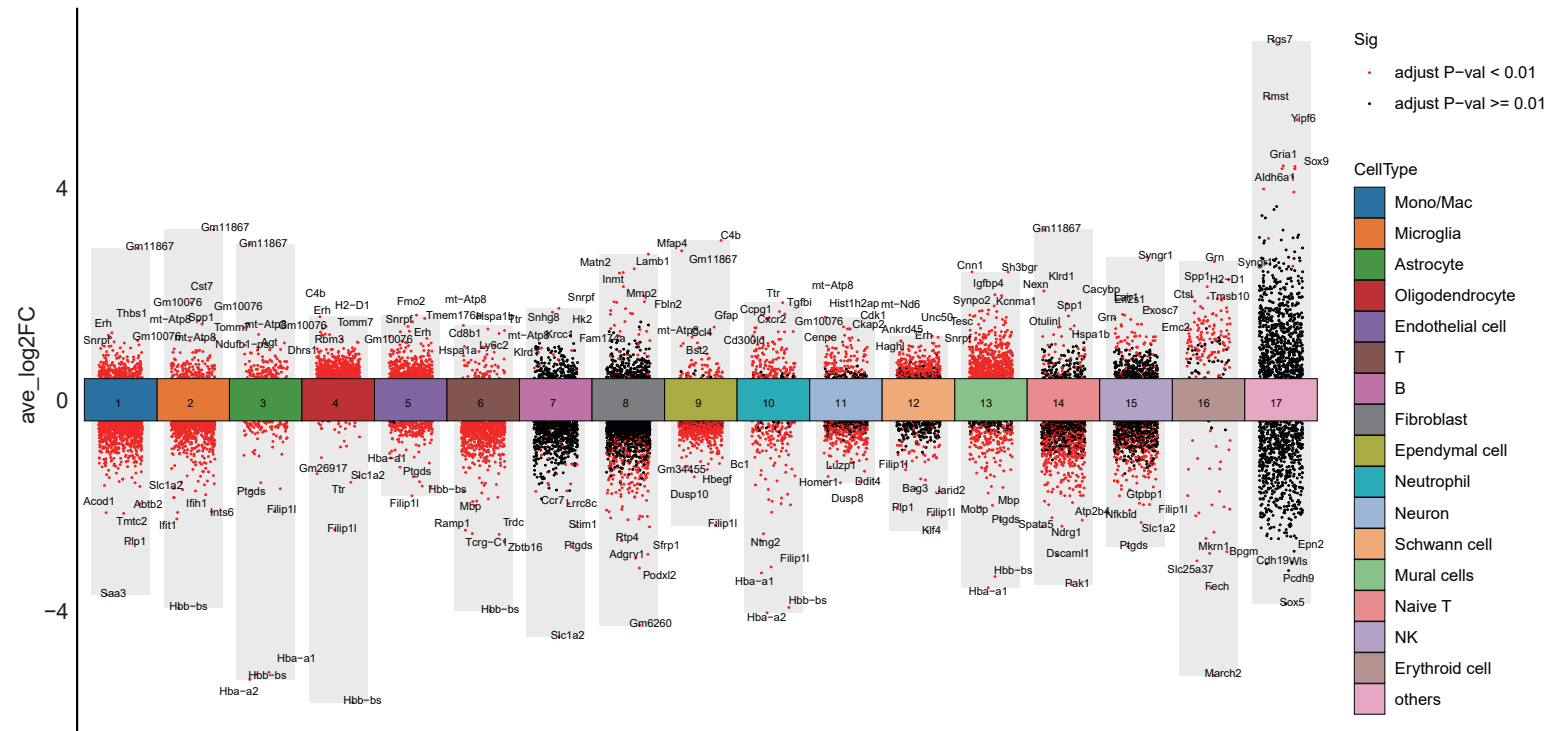

Supplement: Supplementary file 2 — Figure S2: Quality control and differential gene expression analysis of single‐cell RNA sequencing (scRNA‐seq) data. (A) Quality control scRNA‐seq dataset. (B) Volcano plot of differentiated genes in all 16 major cell types of ICH group compared to sham group. [file CNS-31-e70689-s004.pdf]

A

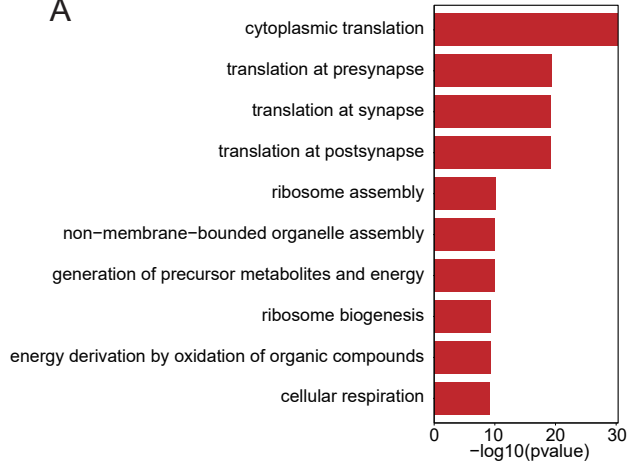

B

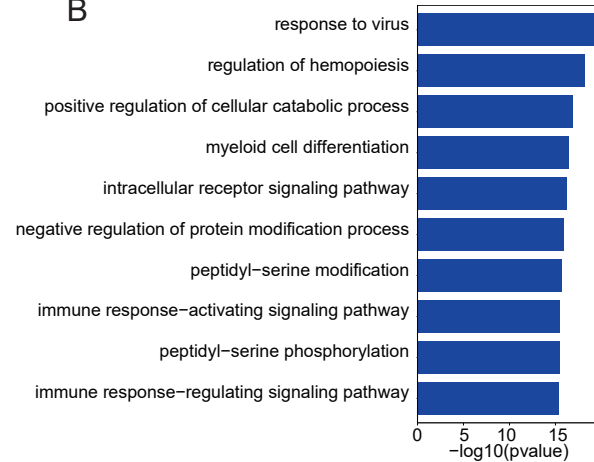

C

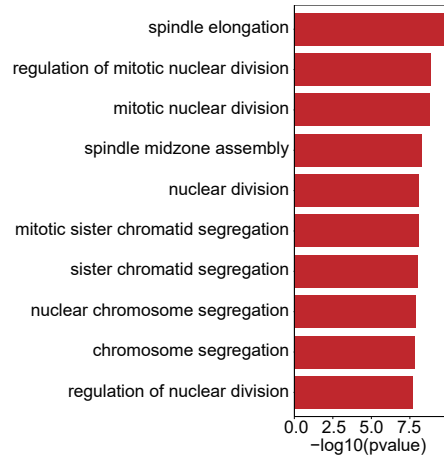

D

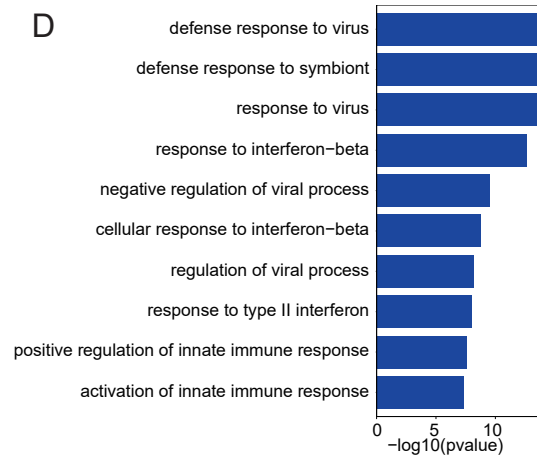

E

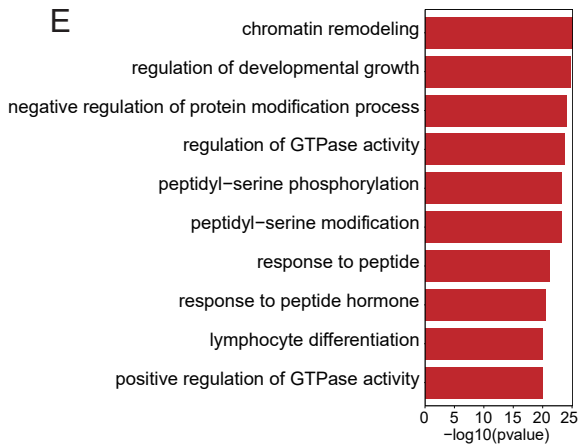

F

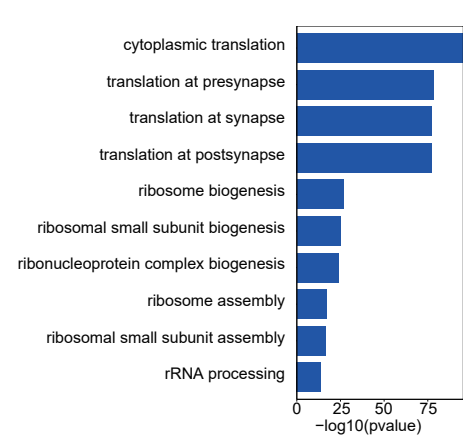

Supplement: Supplementary file 3 — Figure S3: Microglial activation and resolution after acupuncture treatment. (A) Upregulated GO term at microglia in ICH group compared to sham group. (B) Downregulated GO term at microglia in ICH group compared to sham group. (C) Upregulated GO term at microglia in AP‐treated group compared to sham group. (D) Downregulated GO term at microglia in AP‐treated group compared to sham group. (E) Upregulated GO term at microglia subclass MG1 in AP‐treated group compared to ICH group. (F) Downregulated GO term at microglia subclass MG1 in AP‐treated group compared to ICH group. [file CNS-31-e70689-s007.pdf]

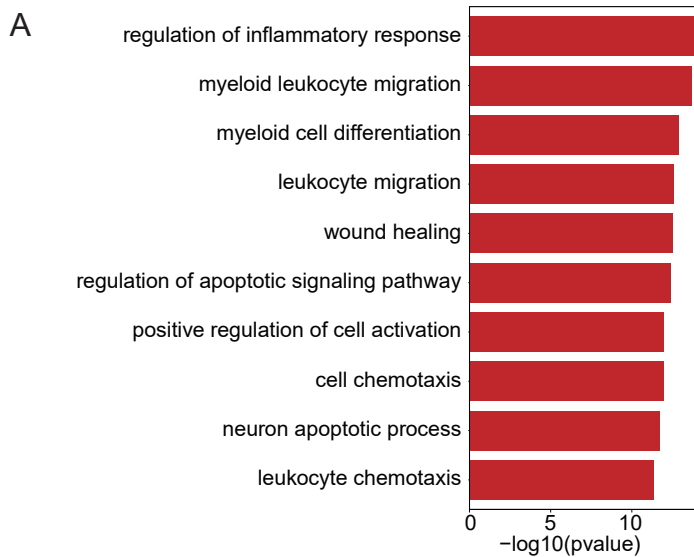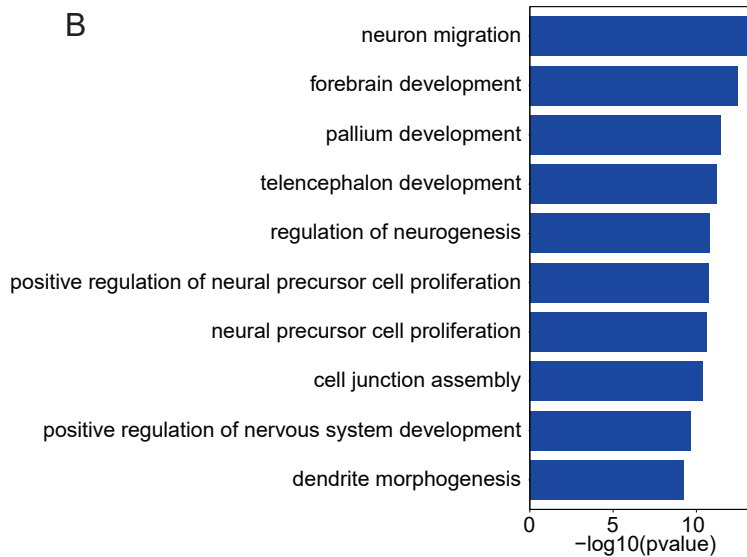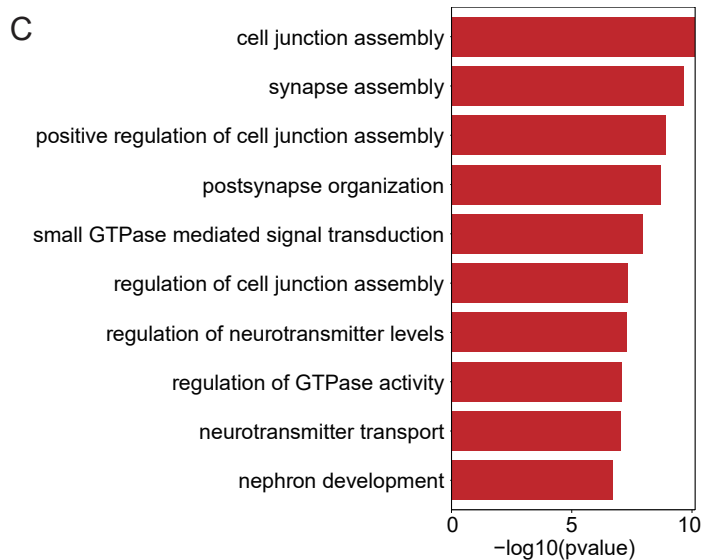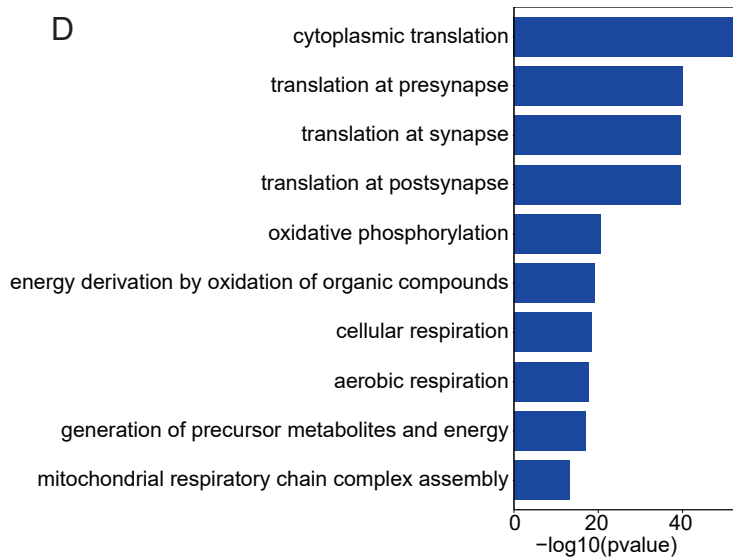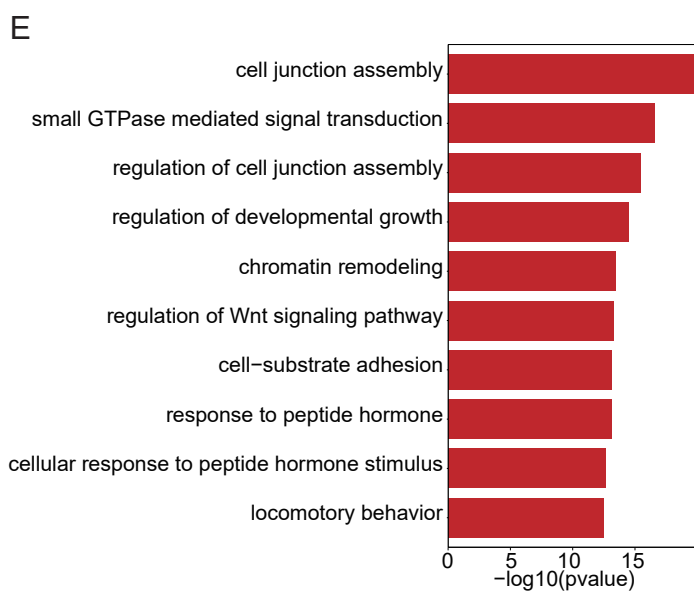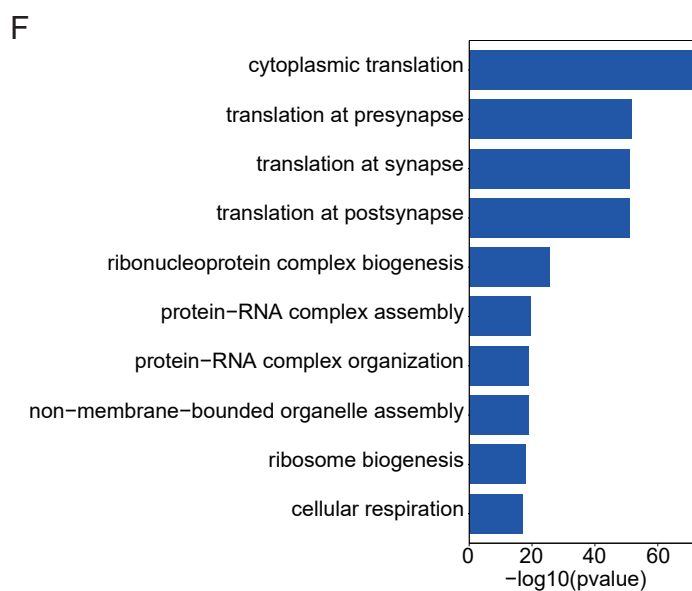

Supplement: Supplementary file 4 — Figure S4: Astrocyte functional plasticity and subtype shifts following acupuncture treatment. (A) Upregulated GO term at astrocyte in ICH group compared to sham group. (B) Downregulated GO term at astrocyte in ICH group compared to sham group. (C) Upregulated GO term at astrocyte in AP‐treated group compared to sham group. (D) Downregulated GO term at astrocyte in AP‐treated group compared to sham group. (E) Upregulated GO term at astrocyte subclass 7 in AP‐treated group compared to ICH group. (F) Downregulated GO term at astrocyte subclass 7 in AP‐treated group compared to ICH group. [file CNS-31-e70689-s003.pdf]

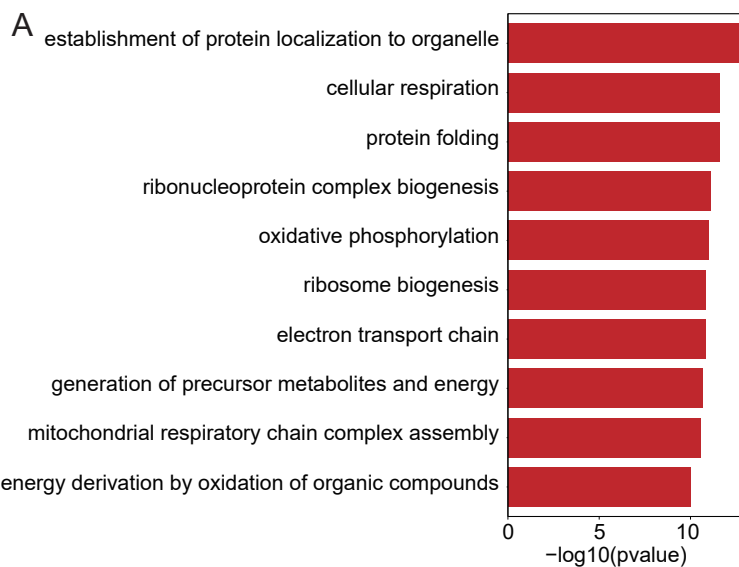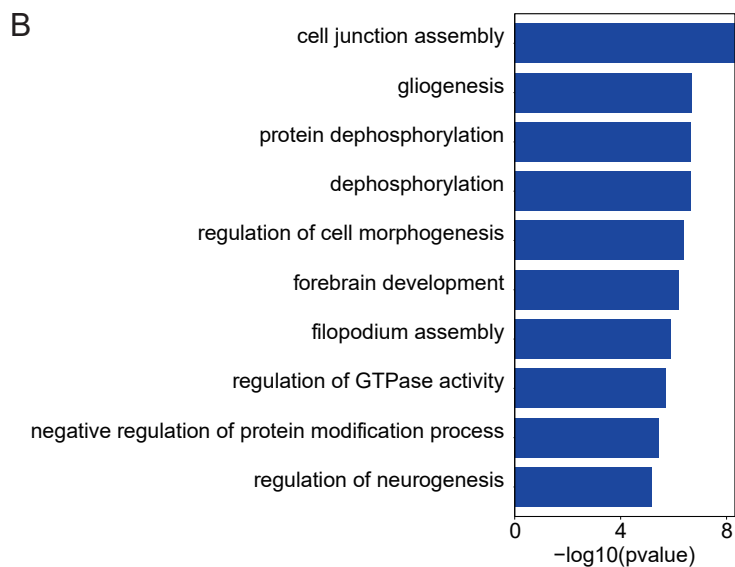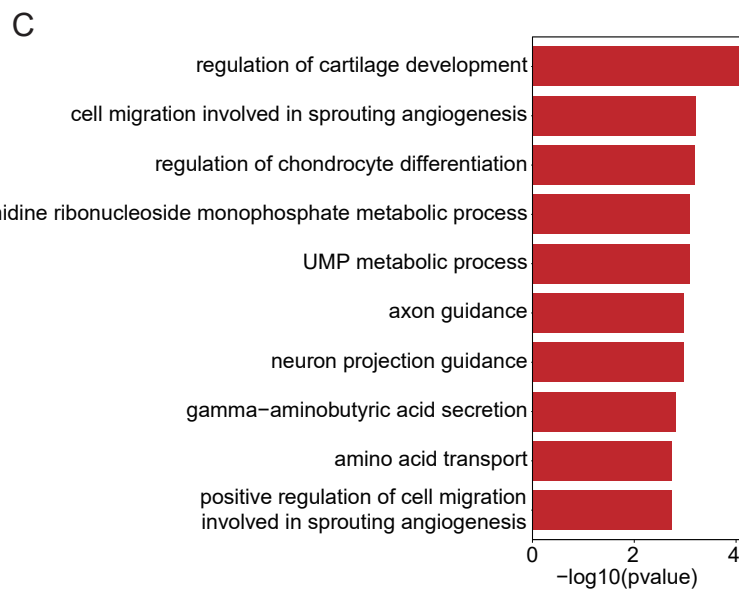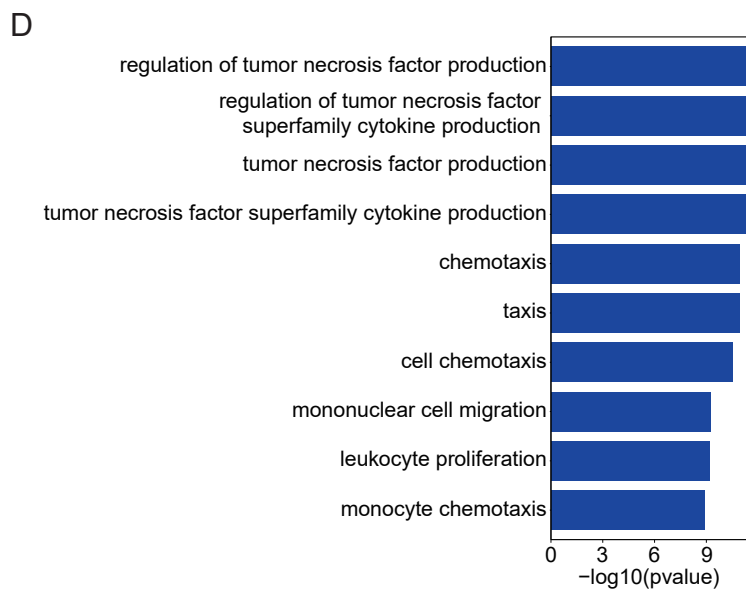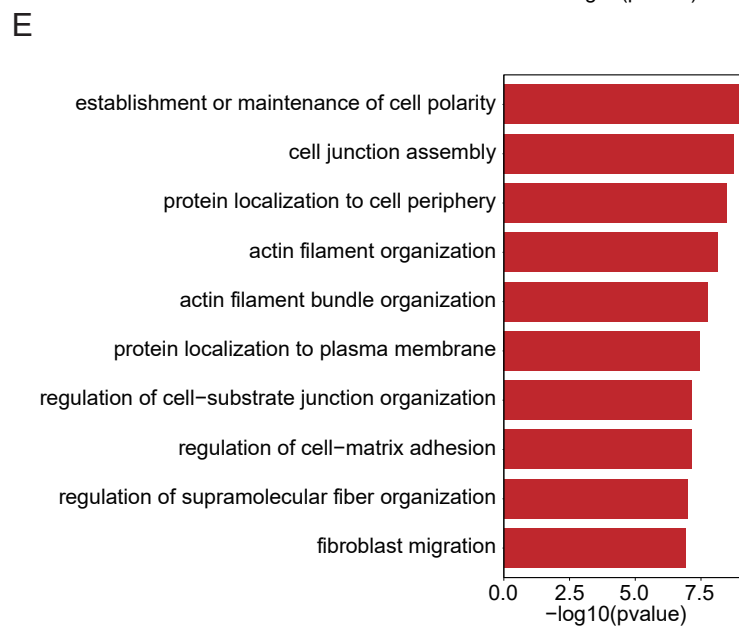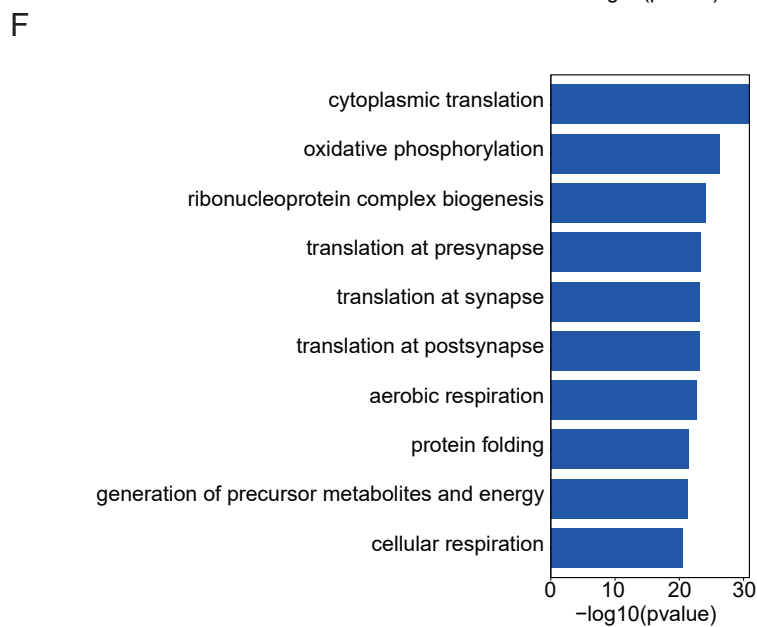

Supplement: Supplementary file 5 — Figure S5: Oligodendrocyte remodeling and myelin regeneration in acupuncture‐treated stroke mice. (A) Upregulated GO term at oligodendrocyte in ICH group compared to sham group. (B) Downregulated GO term at oligodendrocyte in ICH group compared to sham group. (C) Upregulated GO term at oligodendrocyte in AP‐treated group compared to sham group. (D) Downregulated GO term at oligodendrocyte in AP‐treated group compared to sham group. (E) Upregulated GO term at oligodendrocyte subclass 3 in AP‐treated group compared to ICH group. (F) Downregulated GO term at oligodendrocyte subclass 3 in AP‐treated group compared to ICH group. [file CNS-31-e70689-s006.pdf]

A

Differential number of interactions

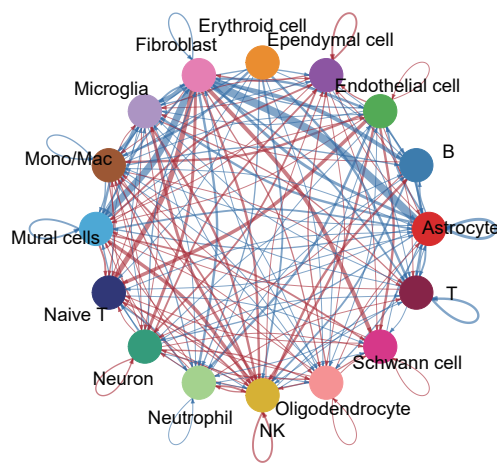

B

Differential interaction strength

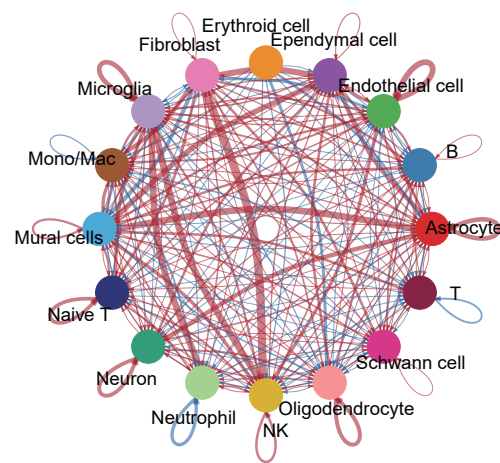

C

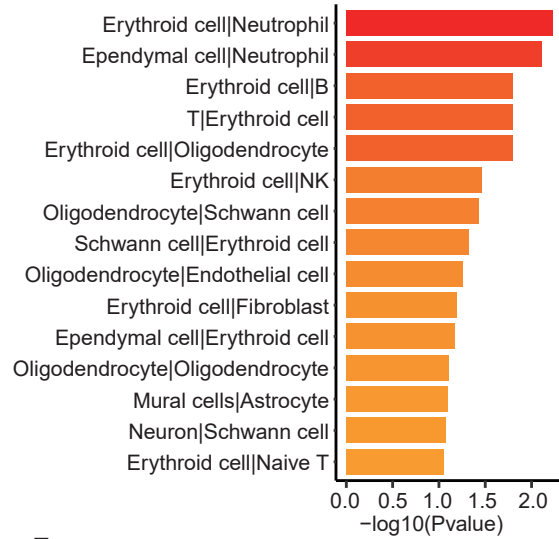

D

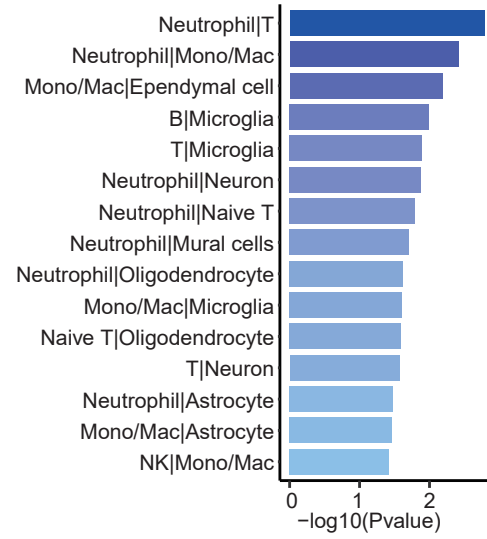

E

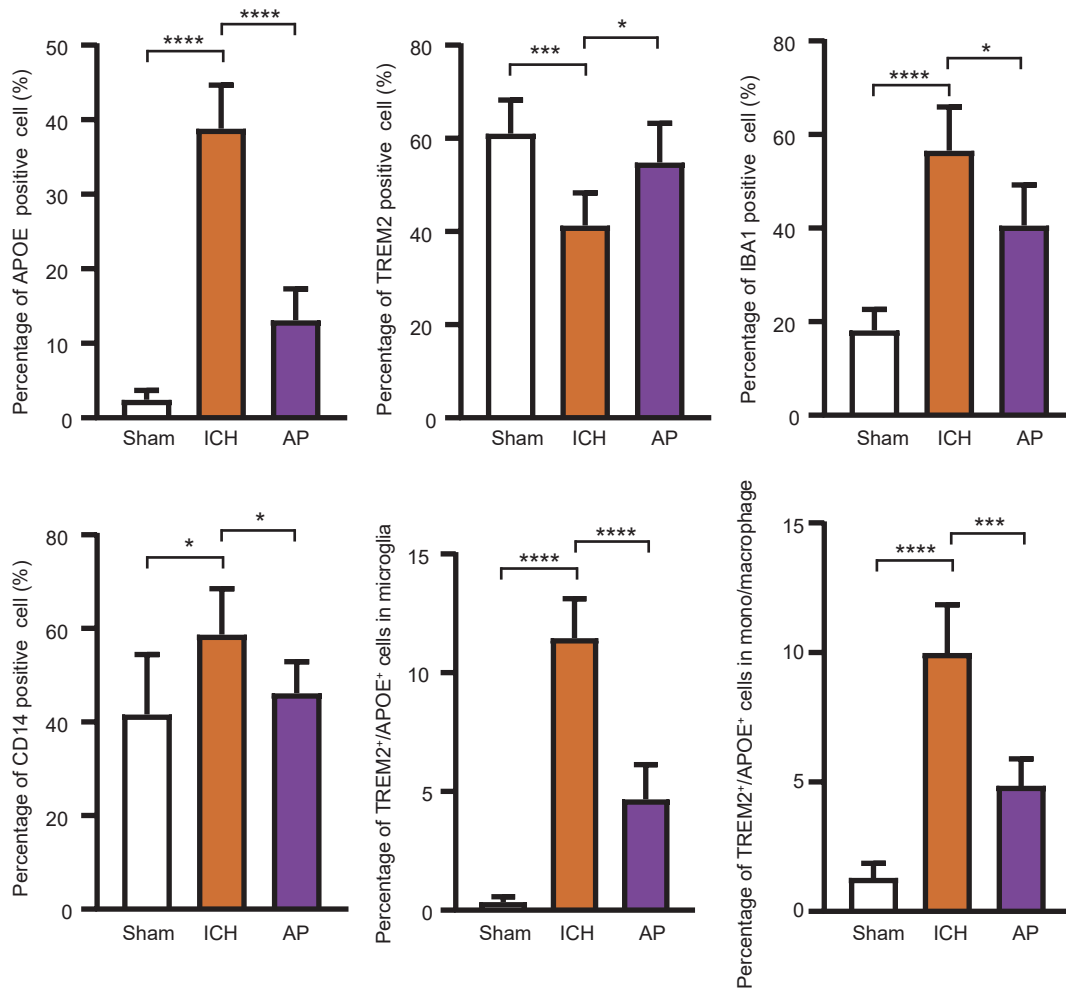

Supplement: Supplementary file 6 — Figure S6: Alterations in cell–cell communication and ligand‐receptor interactions after acupuncture. (A) Differential number of cell–cell interaction in ICH group compared to sham group. (B) Differential cell–cell interaction strength in ICH group compared to sham group. (C) Upregulated cell–cell interaction pairs in ICH group compared to sham group. (D) Downregulated cell–cell interaction pairs in ICH group compared to sham group. (E) Quantification of APOE and TREM2 positive cell in microglia and mono_macrophage. [file CNS-31-e70689-s005.pdf]
